# The iron-regulated small regulatory RNA IsrR modulates expression of genes utilized for dioxygen metabolism and heme synthesis in *Staphylococcus aureus*

**DOI:** 10.1128/mbio.01415-25

**Published:** 2025-10-03

**Authors:** Gustavo Rios-Delgado, Riley McFarlane, Vincent Zheng, Jisun Kim, Dane Parker, Thomas Kehl-Fie, David Lalaouna, Jeffrey M. Boyd

**Affiliations:** 1 Department of Biochemistry and Microbiology, Rutgers, the State University of New Jersey 242612, New Brunswick, New Jersey, USA; 2 Department of Microbiology and Immunology, Carver College of Medicine, University of Iowa, Iowa City, Iowa, USA; 3 Department of Pathology, Immunology and Laboratory Medicine, Center for Immunity and Inflammation, Rutgers New Jersey Medical School, Newark, New Jersey, USA; 4 Université de Strasbourg, CNRS, Architecture et Réactivité de l’ARN, UPR9002, Strasbourg, France; NYU Langone Health, New York, New York, USA

**Keywords:** iron, Fur, *Staphylococcus aureus*, tsr25, IsrR, reactive oxygen species

## Abstract

Bacterial regulatory RNAs (sRNAs) are commonly short non-coding RNAs that function as pleiotropic regulators by post-transcriptionally impacting mRNA stability and/or translation. They play significant roles in bacterial physiology and are typically expressed in response to specific environmental stimuli such as nutrient limitation. The bacterial pathogen *Staphylococcus aureus* faces decreased access to essential metal ions, including iron, in the mammalian host via a process called nutritional immunity. In response to host-mediated iron limitation, *S. aureus* expresses the sRNA IsrR, which coordinates an iron-sparing response by downregulating the expression of mRNAs coding for iron-requiring proteins or processes. Herein, we utilized MS2-Affinity Purification coupled with RNA Sequencing (MAPS) to reveal the *in vivo* IsrR interaction network. Analysis of co-purified RNAs revealed previously unpredicted putative IsrR targets coding for proteins associated with iron-requiring processes. We validated that IsrR directly interacts with nine targets *in vitro*. We demonstrate physiological roles for IsrR in mediating heme biosynthesis, aerobic respiration, and the detoxification of oxygen radicals. These activities are critical for pathogenesis, and this study establishes how *S. aureus* leverages these processes to adapt to iron scarcity, which is commonly encountered in the mammalian host.

**IMPORTANCE:**

*Staphylococcus aureus* causes numerous and varied infections in mammals, making it a significant public health burden and concern. The prevalence of *S. aureus* infections is due to its robust repertoire of virulence factors and its ability to adapt to host microenvironments. Elucidation of the metabolic processes and pathways that promote adaptation to host-promoted stressors provides information about host-pathogen interactions. It could also aid the development of new antimicrobials or unveil treatment and prevention strategies. One common stress bacteria encounter within the mammalian hosts is limited access to iron. In response to iron scarcity, *S. aureus* expresses the regulatory sRNA IsrR. Here, we identified mRNAs that associate with IsrR. We verified that IsrR targets mRNAs that code for proteins involved in aerobic respiration, the metabolism of reactive oxygen species, and heme synthesis. This work provides significant insight into how *S. aureus* responds to host-mediated iron starvation.

## INTRODUCTION

*Staphylococcus aureus* is a human commensal gram-positive bacterium present on 30% of the population, primarily in the anterior nares (1). Upon host entry, *S. aureus* is pathogenic, infecting nearly every organ and causing ailments ranging from minor skin infections to severe diseases such as pneumonia and endocarditis (2). *S. aureus* accounts for most nosocomial-acquired infections in the United States and is a leading cause of non-tuberculosis bacterial infection-related deaths worldwide (3). The prevalence of *S. aureus* as a pathogen is partly due to its wide array of virulence factors, its ability to evade or defend against the host immune system, and metabolic plasticity, which permits the adaptation to different host-encountered stresses (4, 5).

Bacterial non-coding regulatory RNAs (sRNA) are typically short, approximately 50–300 nucleotides long, and have roles in bacterial adaptation to environmental fluctuations, including regulating metabolic pathways, stress responses, virulence, and antibiotic resistance (6). Environmental stimuli trigger their synthesis. Through non-contiguous base pairing, among other mechanisms, they can bind and control mRNA expression by occluding the ribosomal binding site (RBS) on an mRNA, preventing translation initiation, or they can help recruit RNases, leading to mRNA degradation (7). sRNAs can also positively influence expression by revealing a target’s Shine-Dalgarno sequence (SD) or providing stability. High-throughput sequencing has identified numerous sRNAs, but most of them have remained uncharacterized due to the difficulty of identifying their mRNA targets.

To eliminate potential pathogens like *S. aureus,* immune cells, including neutrophils and macrophages, use the oxidative burst (8–10). This defense, catalyzed by NADPH oxidase, generates toxic superoxide, which can undergo dismutation to other reactive oxygen species (11). To overcome oxidative stress*, S. aureus* employs various strategies such as detoxifying enzymes, including heme-containing catalase (KatA), alkyl hydroperoxide reductase (AhpC), two superoxide dismutases (SodA and SodM), and a carotenoid pigment (staphyloxanthin), which serves as an antioxidant (12–14).

*S. aureus* must also overcome the limitation of essential metal ions such as iron (Fe), zinc, and manganese. Host proteins transferrin, lactoferrin, and hemopexin tightly bind metal ions, such as Fe or heme, to restrict their availability to invading bacteria, creating a nutritional immunity (15–17). The principal regulator of iron homeostasis in *S. aureus* is the ferric uptake regulator (Fur) (18). The canonical model for Fur regulation is that Fur is metallated with Fe during iron-replete conditions, has an increased affinity for DNA, and binds to specific sequences known as Fur boxes (19). Fur boxes are typically located in a gene operator, and Fur binding often results in transcriptional repression of a target gene. Upon iron limitation, Fur-mediated repression is alleviated. We recently discovered that Fpa acts as an anti-repressor of Fur to promote the release of Fur from the DNA (20). Upon Fe limitation, the transcription of genes for several Fe uptake systems is derepressed (21).

*S. aureus* Fur represses the transcription of *isrR*, which codes for an sRNA expressed in a low-iron environment. IsrR binds directly to the *fdhA*, *gltB*, *miaB*, *acnA* (*citB*), *sdhC*, *citZ*, *mqo*, *citM,* and *ccpE* transcripts (22–25). Experimental data suggest that IsrR also associates with the *katA*, *rocF*, *narG*, and *nasD* transcripts, but direct experimental evidence is needed (23, 26). These genes code for Fe-requiring proteins or function in Fe-requiring metabolic pathways. We hypothesize that IsrR mediates an iron-sparing response by repressing the expression of non-essential Fe-requiring proteins. IsrR is required for full virulence in murine septicemia and acute pneumonia models (22, 23).

Since the discovery of IsrR, *in silico* analyses have predicted multiple targets, of which nine have been experimentally validated (22–26). Although prediction algorithms can help identify targets and especially interaction sites between sRNA and target RNA, they cannot integrate all factors of base-pairing *in vivo;* therefore, additional approaches have been developed to isolate sRNA-mRNA complexes *in vivo* to reveal mRNA targets (27). One of these approaches is MS2-Affinity Purification coupled with RNA Sequencing (MAPS), where an MS2-tagged sRNA is used to copurify mRNAs that associate with the sRNA *in vivo*, which are later identified and quantified (28, 29). To gain a detailed view into the role of IsrR in the adaptation of *S. aureus* to Fe starvation, we conducted MAPS to help reveal the IsrR targetome. IsrR significantly bound 32 mRNA targets, of which most were not previously predicted through published *in silico* studies (26). We demonstrate direct roles for IsrR in regulating heme synthesis, dioxygen respiration, and the oxidative stress response.

## RESULTS

### MS2-affinity purification, followed by RNA sequencing (MAPS), reveals the IsrR targetome

To gain insight into the IsrR targetome, we conducted MS2-affinity purification coupled with RNA sequencing (MAPS) to identify IsrR-interacting RNAs and quantify these interactions. MAPS allows for the isolation of sRNA-mRNA complexes by tagging an sRNA with the MS2 RNA aptamer, which specifically binds to the MS2 capsid protein (28). This highly specific interaction allows for the affinity copurification of the sRNA-bound mRNAs, which are revealed by RNA sequencing (28).

The MS2 sequence was chromosomally fused to the 5’ end of the *isrR* gene, allowing the synthesis of the chimeric MS2-IsrR in the *S. aureus* HG001 strain from the endogenous promoter. Its expression was triggered using two types of iron-depleted media: (i) brain heart infusion (BHI) broth containing 250 µM of the cell membrane permeable divalent metal chelator 2,2-dipyridyl (DIP), and (ii) Chelex-treated RPMI (NRPMI) supplemented with Mg and Ca. The Δ*isrR* mutant was used as a control. Northern blot analysis confirmed that the MS2-IsrR was expressed and enriched in the elution fraction (Fig. S1). After normalizing read counts, we determined the enrichment of putative RNA-binding partners by comparing the number of reads obtained from the MS2-*isrR* strain and the Δ*isrR* mutant (control). The significant interacting IsrR partners (fold change >2, *P*-value < 0.05) are listed in Tables 1 and 2. Surprisingly, we only enriched one validated IsrR target (*acnA*) (22, 24). Of the 32 significantly enriched mRNA targets, 24 correspond to genes with described functions, and only the *hemA*, *sodM,* and SAUSA300_0872 transcripts were previously predicted to interact with IsrR (23, 26). The putative mRNA targets span multiple cellular processes, including the TCA cycle (*acnA*), heme synthesis, modification, and cytochrome maturation (*hemA, ctaB,* and *ctaM*), cellular respiration (*cydA, cydB,* and *ndhC*), and the oxidative stress response (*sodM*). Additional targets related to pathogenesis (*spa, scb, sbi, efb,* and *lukH*), osmotic stress tolerance (*betA*, *betB*, and *cudC*), and purine synthesis (*purK*, *purE*, and *purQ*) were identified. Many of the putative interacting targets code for proteins that require Fe or are involved in iron-requiring processes, aligning with the described iron-sparing function of IsrR (22, 23, 26).

**TABLE 1:** RNAs that significantly associated with IsrR using MAPS with fold change greater than 2 after growth in Chelex-treated RPMI^*a*^

Gene	Function	Fold enrichment
*betB*	Glycine betaine metabolism	5.8
***ctaM***	**Terminal oxidase maturation**	**5.2**
***ctaB***	**Heme synthesis**	**4.8**
**SAUSA300_0872**	**Unknown**	**4.8**
SAUSA300_2544	Unknown	3.9
*betA*	choline dehydrogenase	3.9
*ald1*	Alanine dehydrogenase	3.0
***acnA***	**Aconitase**	**2.7**
*scb*	Complement inhibitor	2.7
*efb*	Fibrinogen-binding protein	2.6
*cudT*	choline/carnitine/betaine transporter	2.5
*queH*	Epoxyqueuosine reductase	2.5
*purK*	Purine synthesis	2.3
SAUSA300_1334	Unknown	2.3
**SAUSA300_1310**	**Unknown**	**2.3**
*purE*	phosphoribosylaminoimidazole carboxylase	2.3
*cydB*	Cytochrome oxidase	2.3
*ndhC*	NADH dehydrogenase	2.2
SAUSA300_2547	Unknown	2.2
SAUSA300_1831	Unknown	2.2
*sodM*	Superoxide dismutase	2.2
SAUSA300_0432	Unknown	2.2
SAUSA300_1329	amino acid permease	2.1
*lukH*	Bicomponent leukocidin	2.1
*cydA*	Cytochrome oxidase	2.1
*glcA*	Glucose PTS system	2.1
*hemA*	Heme synthesis	2.1
SAUSA300_1620	Unknown	2.0
*sbi*	IgG-binding protein	2.0
*purQ*	Phosphoribosylformylglycinamidine synthase	2.0

^*a*^ All enrichments had a *P*-value of <0.002 as determined by DESeq2 analysis. Transcripts indicated in bold were enriched in both MAPS experiments.

**TABLE 2:** RNAs that significantly associated with IsrR using MAPS with fold change greater than 2 after growth in BHI medium containing 2,2-dipyridyl^*a*^

Gene	Function	Fold enrichment
*spa*	Immunoglobulin G-binding protein A	3.8
***acnA***	**Aconitase**	**3.2**
**SAUSA300_0872**	**Unknown**	**3.1**
**SAUSA300_1310**	**Unknown**	**2.9**
*murG*	Peptidoglycan synthesis	2.2
***ctaM***	**Terminal oxidase maturation**	**2.2**
***ctaB***	**Heme synthesis**	**2.0**

^*a*^ All enrichments had a *P*-value of <0.002 as determined by DESeq2 analysis; 250 µM 2,2-dipyridyl was used. Transcripts indicated in bold were enriched in both MAPS experiments.

### IsrR represses aerobic respiration

IsrR impacts central metabolism by repressing the expression of genes coding for the TCA cycle. Downregulation of the TCA cycle would decrease the number of reducing equivalents generated, which are often used to reduce dioxygen using the respiratory chain during aerobic growth. Our MAPS analysis found that IsrR co-purified with mRNAs coding for dioxygen respiration enzymes, including NADH dehydrogenase (NdhC) and the Fe-requiring terminal oxidase cytochrome *bd* (CydAB).

**Fig 1:**
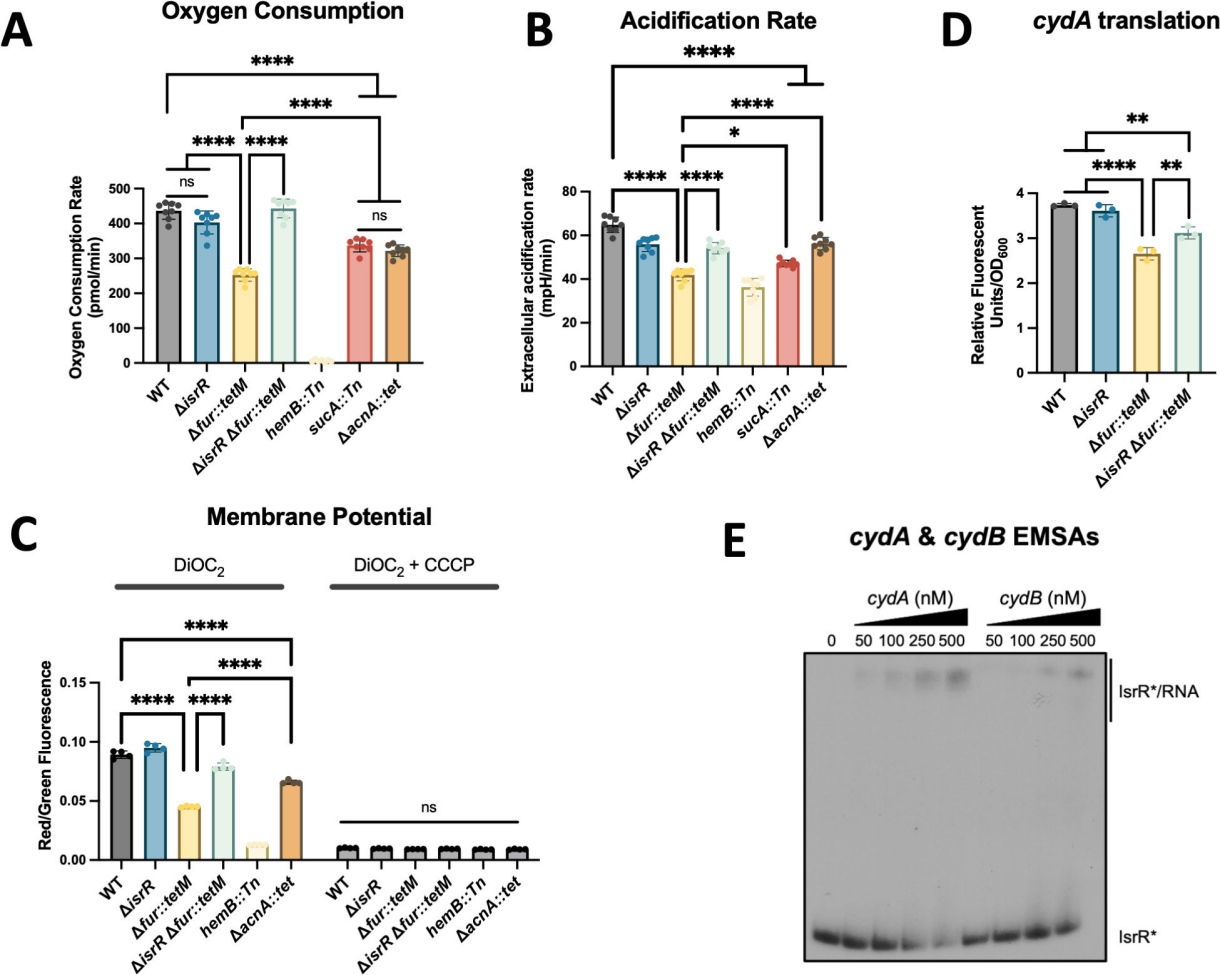
IsrR represses dioxygen respiration. (**A**) Dioxygen consumption rate (OCR) was determined in the wild type (WT; JMB1100), Δ*isrR* (JMB11292), Δ*fur::tet* (JMB10842), Δ*isrR* Δ*fur::tet* (JMB11293), *sucA::Tn* (JMB14125), Δ*acnA::tet* (JMB8563), and *hemB::Tn* (JMB4536) strains cultured in TSB after 34 min incubation at 37°C. (**B**) Extracellular acidification rates of strains in panel A after 90 min of incubation at 37°C. (**C**) Membrane potentials of the WT, Δ*isrR,* Δ*fur,* Δ*isrR* Δ*fur::tet,* Δ*acnA::tet,* and *hemB* strains were measured using the fluorescent dye 3’3’-diethyloxacarbocyanine iodide (DiOC_2_) with and without membrane decoupler carbonyl cyanide m-chlorophenylhydrazone (CCCP). (**D**) Relative fluorescence of the WT, Δ*isrR,* Δ*fur,* and Δ*isrR* Δ*fur* strains containing the pOS_P_lgt__*cydA_gfp* translational reporter after culture in TSB-Cm media. (**E**) An electrophoretic mobility shift assay (EMSA) using 5’ radiolabeled IsrR (*) incubated with increasing concentrations (0, 50, 100, 250, and 500 nM) of *cydA* (−42 to +781) and *cydB* (−172 to +725) transcripts. Data in panels A to D are reported as the average, and error bars indicate standard deviation (A and B, *n* = 8; C, *n* = 4; D, *n* = 3). An ordinary one-way ANOVA (A, B, and D) or two-way ANOVA (C) , followed by Tukey’s multiple comparisons test, was used to analyze the data. *P-value < 0.05, **P-value < 0.01, ****P-value < 0.0001.

**Fig 2:**
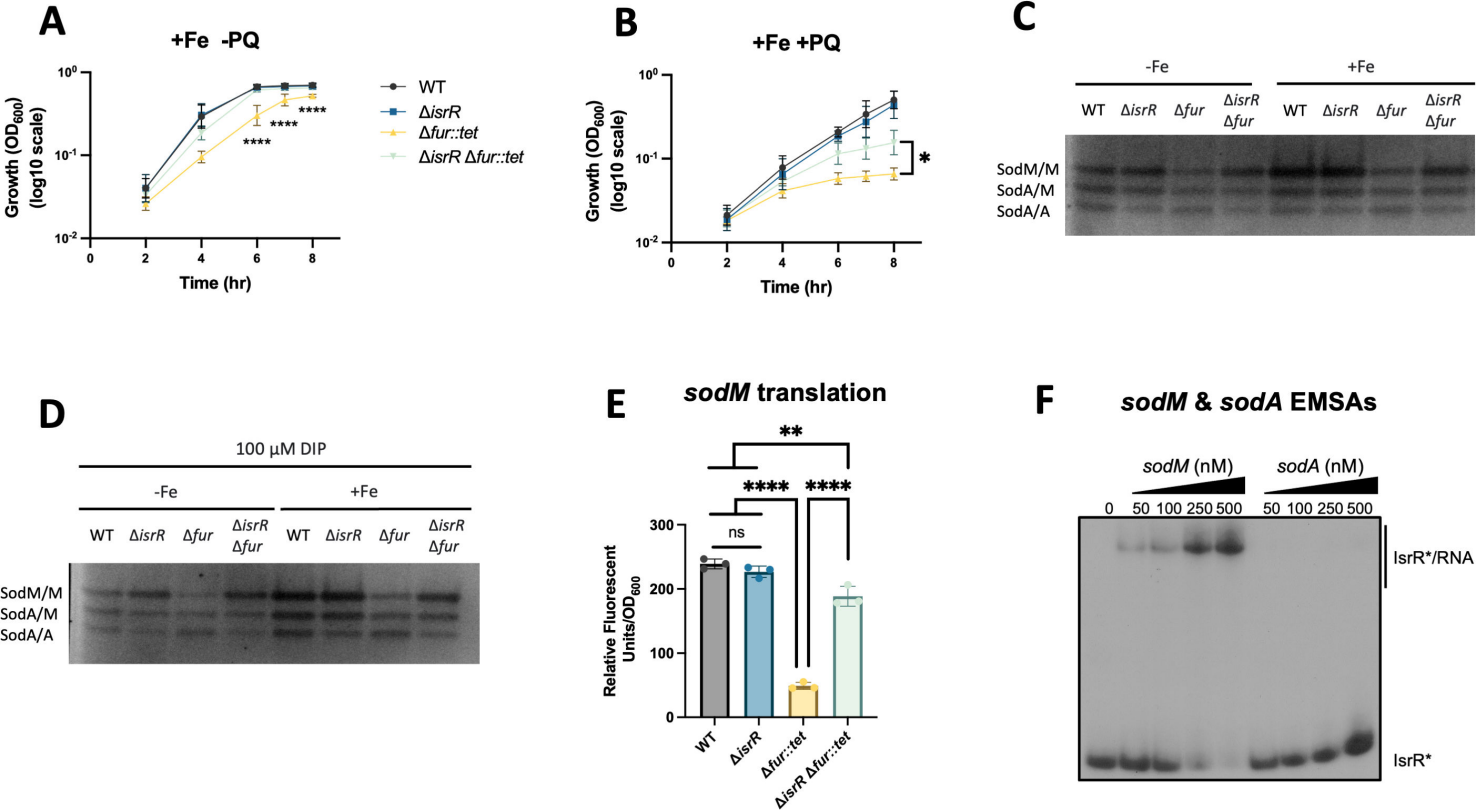
IsrR represses Fe-utilizing superoxide dismutase SodM. Growth of wild type (JMB1100), Δ*isrR* (JMB11292), Δ*fur::tet* (JMB10842), and Δ*isrR* Δ*fur::tet* (JMB11293) cultured in NRPMI supplemented with Mg, Ca, Zn, and Fe (**A**) and with the addition of 0.1 µM paraquat (PQ) (**B**). (**C**) Zymography was used to examine superoxide dismutase activity in the WT, Δ*isrR,* Δ*fur,* and Δ*isrR* Δ*fur* strains after culture in NRPMI medium supplemented with Mg, Ca, and Zn with or without 1 µM FeSO_4_. (**D**) Superoxide dismutase activity gel using the same strains as in panel C cultured with 100 µM 2,2′-dipyridyl (DIP) with or without 1 µM FeSO_4_. For panels C and D, the top band corresponds to the SodM homodimer, the middle band to SodA-SodM heterodimer, and the bottom band corresponds to the SodA homodimer. (**E**) Relative fluorescence of the WT, Δ*isrR,* Δ*fur,* and Δ*isrR* Δ*fur* strains containing the pOS_P_lgt__*sodM_gfp* translational reporter after culture in TSB-Cm media. (**F**) An electrophoretic mobility shift assay (EMSA) using 5’ radiolabeled IsrR (*) incubated with increasing concentrations (0, 50, 100, 250, and 500 nM) of *sodM* (full length, from −28 to +651) and *sodA* (full length, from −28 to +600) transcripts. For panels A, B, and E, data represent experimental averages, and error bars indicate standard deviations (A and B, *n* = 7; E, *n* = 3), but in some cases, error bars are smaller than the symbols used. An ordinary one-way ANOVA, followed by Tukey’s multiple comparisons test, was used to analyze the data in panels A, B, and E. *P-value < 0.05,**P-value < 0.01, ****P-value < 0.0001. For panels A and B, the comparisons between the Δ*fur* and Δ*isrR* Δ*fur* strains are displayed.

We tested the hypothesis that IsrR impacts dioxygen respiration. We quantified the dioxygen consumption rate (OCR) of the WT, Δ*fur::tetM* (Δ*fur*)*,* Δ*isrR,* and Δ*isrR* Δ*fur* strains after culture in Fe-replete tryptic soy broth (TSB) medium, resulting in *isrR* repression in the WT and expression in the Δ*fur* strain (22). Heme is a necessary cofactor for the staphylococcal terminal oxidases CydAB and QoxABCD (30). We used a heme auxotroph (*hemB::Tn*) as a negative control. The *hemB* mutant had no detectable oxygen consumption, verifying respiration is the primary consumer of dioxygen under the growth conditions examined (Fig. 1A and 2A). The Δ*isrR* strain had a comparable OCR to the WT, whereas the rate in the Δ*fur* strain was significantly lower (Fig. 1A). The OCR of the Δ*fur* Δ*isrR* strain was comparable with that of the WT strain, suggesting that increased *isrR* expression in the Δ*fur* mutant was responsible for the decreased oxygen consumption. We noted a similar trend when monitoring the medium acidification rate after culture in Fe-replete TSB. The acidification rate was significantly decreased in the Δ*fur* strain compared with the WT or Δ*isrR* strains but restored to WT levels in the Δ*isrR* Δ*fur* mutant (Fig. 1; Fig. S2B).

To confirm IsrR was mediating the decreased oxygen consumption in the Δ*fur* mutant, we genetically complemented the Δ*isrR* Δ*fur* double mutant by integrating *isrR* at a secondary location using the pLL39 episome. The pLL39 vector encodes for tetracycline resistance; therefore, instead of the Δ*fur::tetM* allele, we used the previously reported null *fur_E11stop_* (fur*) allele that is linked to the *proC::Tn* transposon (20). As predicted, the fur* proC::Tn pLL39 strain showed significantly decreased OCR compared with the parent (Fig. S3A). The OCR of the fur* strain was partially restored in the ΔisrR fur* strain and could be genetically complemented (Fig. S3A).

We also quantified the OCR in the Δ*acnA::tet* and *sucA::Tn* strains, which lack the TCA cycle enzymes aconitase and 2-oxoglutarate dehydrogenase, respectively. Both mutants had reduced OCR compared with the WT, but not to the level of the Δ*fur* mutant (Fig. 1; Fig. S2A). The finding that the OCR of the Δ*fur* strain was decreased compared with the TCA cycle mutants suggests the impact of IsrR on respiration is not fully explained by IsrR-mediated TCA cycle repression.

A decreased rate of dioxygen respiration should result in a decreased electrochemical gradient. We monitored the membrane potential using the fluorescent dye 3’3’-diethyloxacarbocyanine iodide (DiOC_2_). A robust membrane potential promotes DiOC_2_ accumulation and association intracellularly, causing a shift from emitting green to red fluorescence. A strain with a weaker membrane potential will accumulate less dye and have a decreased ratio of red to green fluorescence (31). We monitored the membrane potential in the WT, Δ*fur,* Δ*isrR,* Δ*isrR* Δ*fur,* Δ*acnA::tet,* and *hemB::Tn* strains using DiOC_2_ after culture in Fe-replete TSB medium. Intracellular DiOC_2_ accumulation mimicked what we observed for oxygen consumption. The WT and Δ*isrR* strains accumulated red-fluorescing DiOC_2_, suggesting a robust membrane potential (Fig. 1C). The Δ*fur* strain displayed a decreased ratio of red to green fluorescence, indicating a decreased membrane potential. The ratio of red to green fluorescence was increased in the Δ*isrR* Δ*fur* strain compared with the Δ*fur* strain. The membrane potential phenotype of the ΔisrR fur* strain could be genetically complemented, demonstrating a role for IsrR in decreasing the formation of an electrochemical gradient in a null *fur* strain (Fig. 1; Fig. S3B). As a positive control for loss of membrane potential, we added carbonyl cyanide m-chlorophenylhydrazone (CCCP), which dissipates the transmembrane electric potential and results in decreased ratio of red/green fluorescence (Fig. 1; Fig. S3B).

In addition to decreased oxygen consumption, medium acidification rate, and membrane potential, we demonstrate that a Δ*fur* mutant has decreased growth yield compared with the wild type (Fig. S4). The growth rate and yield of the Δ*fur* mutant were partially restored in the Δ*isrR* Δ*fur* strain.

The *cydA* and *cydB* genes code for a terminal oxidase, are co-transcribed, and were enriched in our MAPS experiments (32). We identified potential IsrR binding sites overlapping the ribosomal binding site of *cydA* and upstream of *cydB* (Fig. S9A and B). To begin examining how *isrR* was impacting the translation of *cydA*, we constructed a translational reporter fusion where the 5’ untranslated region of *cydA* and the initial two codons were fused with a promoterless *gfp*. We moved the reporter construct to the WT, Δ*fur,* Δ*isrR,* and Δ*isrR* Δ*fur* strains and quantified Gfp fluorescence after culture in TSB. The WT and Δ*isrR* strains produced similar levels of Gfp. The *fur* mutant had decreased Gfp production, which was partially but significantly restored in the Δ*isrR* Δ*fur* strain, suggesting that IsrR negatively impacts *cydAB* translation (Fig. 1D).

We performed electrophoretic mobility shift assays (EMSA) using radiolabeled IsrR to confirm the direct interaction with the *cydA* and *cydB* mRNA transcripts. IsrR bound to the *cydA* and *cydB* transcripts with apparent affinities of <250 nM and <500 nM, respectively (Fig. 1E). These results are consistent with a model where, upon alleviating Fur repression of *isrR*, IsrR is expressed and binds to the *cydAB* transcript, resulting in decreased translation and decreased respiration.

### IsrR represses expression of the cambialistic superoxide dismutase SodM

The aerobic respiratory chain is a primary producer of superoxide in dioxygen-respiring cells (33, 34). *S. aureus* combats superoxide stress by expressing two metal-containing superoxide dismutases (SOD) (SodA and SodM) (35, 36). SodA is strictly Mn-dependent and the primary SOD when Mn is available (14). SodM is induced in response to Mn limitation mediated by calprotectin, an effector of nutritional immunity. Although capable of using both Mn and Fe as its metal cofactor, in Mn-limited environments, SodM utilizes Fe (14). The MAPS analysis determined that the *sodM* transcript co-purified with IsrR. IntaRNA analysis revealed a potential IsrR binding site overlapping the *sodM* SD (Fig. S10A).

To confirm the role of *isrR* in impacting the response against superoxide, we cultured the WT, Δ*fur*, Δ*isrR*, and Δ*isrR* Δ*fur* strains in NRPMI supplemented with 1% Casamino acids, MgCl_2_, CaCl_2_, ZnSO_4_, and FeSO_4_ in the presence or absence of superoxide-generating paraquat (PQ). In the absence of PQ, the WT, Δ*isrR*, and Δ*isrR* Δ*fur* strains grew comparably, whereas the Δ*fur* displayed a growth defect (Fig. 2A). It has been reported that a *fur* mutant has reduced growth compared with the WT, and it was hypothesized that this is partially due to an inability to effectively metabolize oxidative stress (18). In the presence of 0.1 µM of PQ, the Δ*isrR* grew comparably with the WT strain, whereas the Δ*fur* mutant was hypersensitive (Fig. 2B). The drastic growth defect of the Δ*fur* strain was partially recovered in the Δ*isrR* Δ*fur* strain, suggesting a role for IsrR in the response to superoxide stress.

We tested the hypothesis that the hypersensitivity of the Δ*fur* mutant to PQ was due to a change in superoxide dismutase (SOD) expression. We monitored the activities of SodA and SodM in the WT, Δ*fur*, Δ*isrR*, and Δ*isrR* Δ*fur* strains during growth in the presence and absence of exogenously supplemented Fe. After growth with Fe, all strains had increased SodM activity (Fig. 2; Fig. S5). With or without Fe supplementation, the Δ*isrR* strain had comparable SodM activity with the WT. The Δ*fur* mutant had decreased SodM activity, exacerbated by Fe deficiency. The Δ*isrR* Δ*fur* strain had increased SodM activity compared with the Δ*fur* strain, suggesting that IsrR impacts SodM activity in the absence of Fur.

We expected that growth with low Fe, which alleviates Fur-mediated repression of *isrR*, would decrease SodM activity in the WT but not the Δ*isrR*. Since the WT and Δ*isrR* strains had comparable SodM activity, we interpret it to mean that we still had Fur-mediated repression of *isrR* in the WT and deduced that the growth medium was not sufficiently starved for Fe. We repeated the SOD activity assays with the addition of DIP to limit Fe availability. In cultures with DIP, all strains had decreased SodM activity, which was relieved by adding Fe (Fig. 2; Fig. 5). The Δ*fur* strain had decreased SodM activity after culture with DIP and DIP + Fe, which was rescued upon introducing the Δ*isrR* allele. Adding DIP to starve the medium for Fe further increased SodM activity in the Δ*isrR* strain compared with the WT (Fig. 2D). These data support a model where, upon sufficient Fe depletion, *isrR* is expressed in the WT strain, which represses the expression of SodM as part of the Fe-sparing response.

We tested the hypothesis that IsrR decreases the production of SodM by interacting with the mRNA transcript. The predicted IsrR-*sodM* mRNA interaction overlapped the *sodM* SD, suggesting that the interaction negatively impacted *sodM* translation by RBS occlusion. To test the impact of IsrR on *sodM* translation, we constructed a translational reporter using the *sodM* 5’ UTR to drive the transcription and translation of *gfp* (pOS_P*_lgt_*_*sodM_gfp*). The reporter construct was transduced into the WT, Δ*fur*, Δ*isrR*, and Δ*isrR* Δ*fur* strains, and fluorescence was quantified. The WT and Δ*isrR* strains had comparable Gfp production (Fig. 2E). The Δ*fur* mutant had decreased *sodM* translation, which was recovered in the Δ*isrR* Δ*fur* strain (Fig. 2E).

We performed EMSAs to test the hypothesis that IsrR directly interacts with the *sodM* mRNA. We also conducted EMSA using the *sodA* mRNA as a negative control to demonstrate specificity since *sodA* and *sodM* 5’UTRs are similar. Adding *sodM* mRNA but not *sodA* mRNA resulted in gel retardation, demonstrating the formation of a complex with IsrR *in vitro* (Fig. 2F). These results are consistent with the hypothesis that under Fe limitation or in the absence of Fur, *isrR* is expressed and interacts with the *sodM* transcript, resulting in decreased translation, decreased SodM activity, and sensitivity to superoxide.

### IsrR represses catalase activity by directly binding to the *katA* mRNA transcript

Hydrogen peroxide (H_2_O_2_) is produced by superoxide dismutase, and H_2_O_2_ can accumulate as oxygen increases, suggesting that in some organisms, the respiratory chain is a source of H_2_O_2_ (37–39). Proteomic and *in silico* analyses by Ganske et al. identified a potential IsrR interaction site in the 5’ UTR of the *katA* transcript. They also demonstrated that an *S. aureus* HG001 *fur* mutant strain was sensitive to H_2_O_2_ (26). Using IntaRNA (40), we identified a putative IsrR interaction site in the *katA* mRNA transcript that spanned from the SD through the initial coding sequence (Fig. S10).

**Fig 3:**
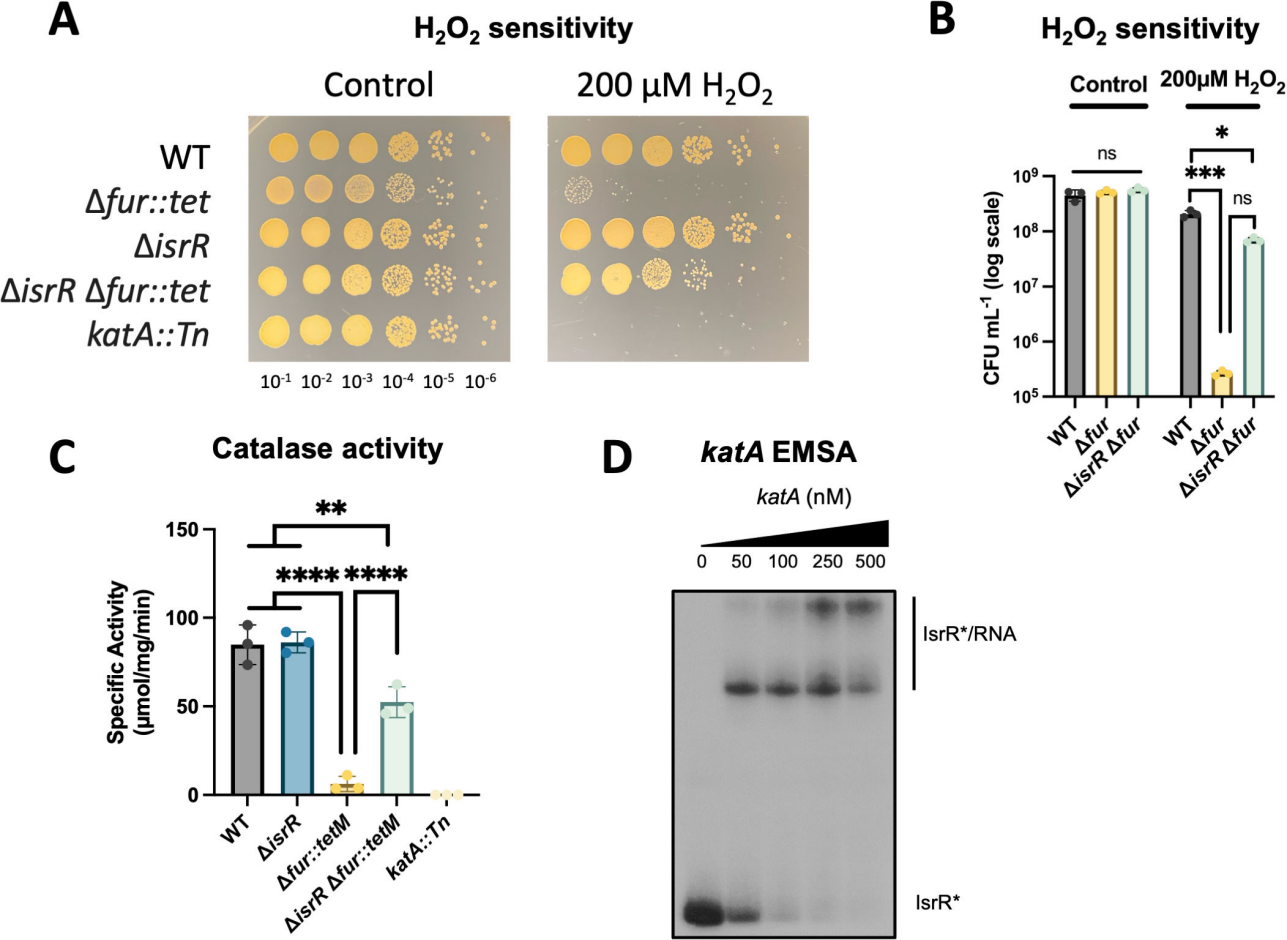
IsrR represses catalase synthesis. (**A**) Hydrogen peroxide sensitivity of wild type (JMB1100), Δ*isrR* (JMB11292), Δ*fur::tet* (JMB10842), Δ*isrR* Δ*fur::tet* (JMB11293), and *katA::Tn* (JMB2078) strains. Bacterial cells were treated with a bolus of H_2_O_2_ or PBS (control). The reaction was quenched with catalase, bacteria were serially diluted, and spot-plated on TSA plates to determine colony-forming units (CFU). The experiment was performed in triplicate, and an image of a representative experiment is displayed. (**B**) Quantification of the CFU mL^−1^ of the wild type, Δ*fur,* and Δ*isrR* Δ*fur* strains in panel A. (C) The specific activity of catalase was quantified in cell-free lysates from the strains in panel A. (D) An electrophoretic mobility shift assay (EMSA) using 5’ radiolabeled IsrR (*) incubated with increasing concentrations (0, 50, 100, 250, and 500 nM) of *katA* RNA transcript (−42 to +392). For panels B and C, bars indicate the average, and errors are displayed as standard deviation (*n* = 3). A two-way ANOVA (B) or ordinary one-way ANOVA (C) followed by Tukey’s multiple comparisons test was used to analyze the data. **P-value < 0.01, ****P-value < 0.0001.

We tested the hypothesis that IsrR impacts *katA* expression in *S. aureus* LAC. We assessed the ability of the WT, Δ*fur*, Δ*isrR*, Δ*isrR* Δ*fur*, and *katA::Tn* strains to survive a challenge with H_2_O_2_. The WT and Δ*isrR* strains fully survived the challenge, and there was no detectable survival of the *katA::Tn* strain, which is consistent with KatA being the primary H_2_O_2_ metabolizing enzyme in *S. aureus* under the conditions examined (Fig. 3A). The Δ*fur* strain was hypersusceptible to H_2_O_2_ killing, and this phenotype was partially recovered in the Δ*isrR* Δ*fur* strain (Fig. 3A). Although statistical analysis showed no significant difference between the Δ*isrR* Δ*fur* strain and the Δ*fur* under H_2_O_2_, there was a 260-fold increase in CFU mL^−1^ of the Δ*isrR* Δ*fur* compared with the Δ*fur* mutant (Fig. 3B).

The genes that code for Fe uptake systems are derepressed in a *fur* mutant, and an abundance of cytosolic Fe could potentiate H_2_O_2_ killing through Fenton reaction radical formation. To address this, we tested the H_2_O_2_ sensitivity of the parent, Δ*isrR* mutant, and the *isrR* complemented strain after culture in TSB with and without 250 µM DIP to induce *isrR* expression in the wild type. After culture in Fe-replete TSB medium, all strains were resistant to H_2_O_2_ challenge (Fig. S6A and B). Upon co-culture with DIP, the wild type was sensitive to H_2_O_2_, whereas the Δ*isrR* strain resisted H_2_O_2_ killing (Fig. S6A and C). Genetic complementation of the Δ*isrR* strain resulted in H_2_O_2_ sensitivity comparable with the wild type, demonstrating *isrR* expression leads to increased sensitivity to H_2_O_2_.

We tested the hypothesis that the sensitivity of the Δ*fur* strain to H_2_O_2_ was due to decreased catalase activity and not to a decrease in another mechanism promoting antioxidant activity (12, 13). We quantified H_2_O_2_ consumption in cell-free lysates of the WT, Δ*fur*, Δ*isrR*, Δ*isrR* Δ*fur*, and *katA::Tn* strains. The consumption of H_2_O_2_ was indistinguishable in the WT and Δ*isrR* strains. The Δ*fur* mutant had nearly undetectable consumption capability, and no H_2_O_2_ was degraded in the *katA::Tn* strain, indicating that KatA was responsible for the H_2_O_2_ consumption under the conditions examined. The low catalase activity of the Δ*fur* strain was partially recovered upon introducing the Δ*isrR* mutation, and this phenotype could be genetically complemented (Fig. 3; Fig. S7).

We examined whether the IsrR-mediated catalase repression could be through direct interaction with the *katA* mRNA transcript. We performed an EMSA using radiolabeled IsrR. We observed two band shifts, confirming the direct interaction between IsrR and *katA* mRNA and suggesting two possible interaction sites (Fig. 3D). These data align with the hypothesis that upon alleviation of Fur-mediated repression of *isrR,* IsrR interacts with the *katA* mRNA transcript, leading to decreased *katA* expression and increased sensitivity to H_2_O_2_.

### IsrR represses heme biosynthesis

Heme is an essential prosthetic group of *S. aureus* terminal oxidases (CydAB and QoxABCD) and catalase (KatA) (30). MAPS analysis revealed IsrR copurified with mRNAs coding for proteins involved in heme synthesis, modification, and cytochrome maturation (HemA, CtaB, and CtaM) (Tables 1 and 2). *S. aureus* synthesizes heme using the enzymes encoded by the *hemAXCDBL, hemEHY*, and *hemN* operons (41). The enzymes from the *hemAXCDBL* operon coordinate the formation of uroporphyrinogen III (UroIII) from glutamyl tRNA, where glutamyl tRNA reductase (HemA/GtrR) catalyzes the first committed step in heme synthesis (41). The enzymes from the *hemEHY* operon coordinate the formation of protoheme IX (heme B) (41). Upon formation of heme B, heme O synthase (CtaB) and heme A synthase (CtaA) catalyze the formation of heme O and heme A, respectively, which are essential for a functional QoxABCD (42). CtaM is required for a functional QoxABCD formation and is predicted to be a potential heme chaperone (42). Using the IntaRNA *in silico* analysis, we identified potential IsrR binding sites in *hemA, ctaM,* and *ctaB* transcripts (Fig. S11). We also found predicted interactions between IsrR and the *hemE* and *ctaA* mRNA transcripts, which were not identified by the MAPS analyses (Fig. S11). IsrR is predicted to interact with the RBS of *ctaB* and *hemA,* and the beginning coding region of the *hemE* and *ctaM* transcripts (Fig. S11).

**Fig 4:**
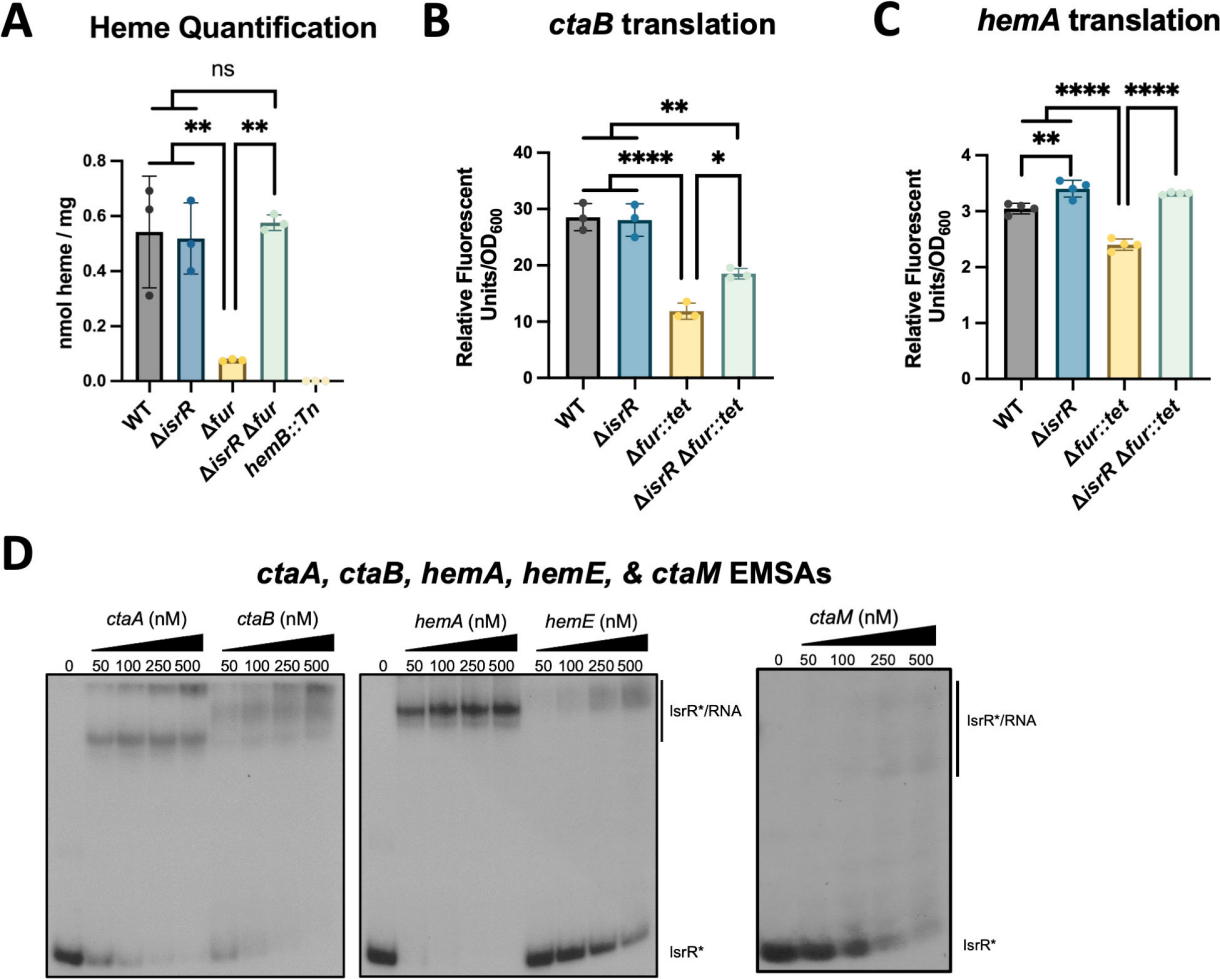
IsrR regulates heme biosynthesis. (**A**) Heme was quantified in the wild type (WT; JMB1100), Δ*isrR* (JMB11292)*,* Δ*fur::tet* (JMB10842)*,* and Δ*isrR* Δ*fur::tet* (JMB11293)*,* and *hemB::Tn* (JMB4536) strains after culture in TSB medium. (**B**) Relative fluorescence in the WT, Δ*isrR*, Δ*fur,* Δ*isrR* Δ*fur* strains containing the pOS_P_lgt__*ctaB_gfp* translational reporter after culture in TSB-Cm media. (**C**) Relative fluorescence of WT, Δ*isrR*, Δ*fur*, Δ*isrR* Δ*fur* strains containing the pOS_P_lgt__*hemA_gfp* translational reporter after culture in TSB-Cm media. (**D**) Electrophoretic mobility shift assays (EMSA) using 5’ end-radiolabeled IsrR (*) incubated with increasing concentrations (0, 50, 100, 250, and 500 nM) of *ctaA* (from −124 to +513)*, ctaB* (from −27 to +751)*, hemA* (from −28 to +691)*, hemE* (from −36 to +797), or *ctaM* (full length, from −207 to +462) RNA transcripts. Data in panels A to C indicate experimental averages, and error bars indicate standard deviation (*n* = 3). An Ordinary one-way ANOVA followed by Tukey’s multiple comparisons test was used to analyze the data. *P-value < 0.05, **P-value < 0.01, ****P-value < 0.0001.

We hypothesized that IsrR interaction with the mRNA transcripts controlling heme synthesis would decrease heme production. We isolated and quantified heme from the WT, Δ*fur,* Δ*isrR,* and Δ*isrR* Δ*fur* strains and included a *hemB::Tn* strain as a negative control. We were able to detect heme in the WT and Δ*isrR* strains but not the *hemB::Tn* strain (Fig. 4A). The heme concentration was greatly decreased in the Δ*fur* strain but increased upon introducing the Δ*isrR* mutation (Fig. 4A). The heme synthesis phenotype of a ΔisrR fur* double mutant could be genetically complemented ( Fig. S8).

To assess the impact of IsrR on the translation of heme biosynthesis transcripts, we constructed *hemA* and *ctaB* translational reporters. The translational reporters contained either the *hemA* or *ctaB* 5’ UTR and their two initial codons cloned in frame with *gfp,* allowing us to monitor the expression of the chimeric protein. The fluorescence attributed to the *ctaB* and *hemA* translational reporters was quantified in the WT, Δ*fur,* Δ*isrR,* and Δ*isrR* Δ*fur* strains. For the *ctaB* reporter, the Δ*isrR* had comparable *ctaB* translation to the WT, whereas the Δ*fur* had decreased *ctaB* expression, which was recovered on the Δ*isrR* Δ*fur* strain (Fig. 4B). The *hemA* reporter had a similar expression pattern*,* suggesting that IsrR is mediating the translational repression of both *ctaB* and *hemA* in the absence of *fur* (Fig. 4C).

We conducted EMSAs to confirm the MAPS data and validate the direct interactions between IsrR and the *hemA, hemE, ctaA*, *ctaB,* or *ctaM* transcripts. Combining IsrR with each RNA retarded the migration of radiolabeled IsrR, suggesting direct binding (Fig. 4D). The IsrR-*ctaM* mRNA interaction resulted in the lowest apparent affinity. In the IsrR-*ctaM* mRNA EMSA, there were two faint bands in the 250 nM and 500 nM lanes at higher molecular weight, and the labeled IsrR* decreases in intensity upon *ctaM* mRNA titration, which supports direct binding. These results are consistent with a model wherein Fe-deprivation results in Fur alleviating *isrR* repression, IsrR interacting with the *hemA, hemE, ctaA, ctaB,* and *ctaM* mRNA transcripts, decreasing expression, and negatively impacting heme production.

## DISCUSSION

In response to iron-limiting conditions, *S. aureus* expresses the sRNA IsrR (22). We initiated this study to better understand the staphylococcal response to Fe-limitation by uncovering the IsrR targetome. *In silico* work by others obtained sets of IsrR putative targets, with each study identifying around 20 mostly distinctive targets, of which most remain unvalidated (23, 26, 32). Although helpful in predicting sRNA function and locating potential interacting mRNAs, the output of *in silico* approaches should be carefully considered since they cannot integrate all factors influencing base pairing *in vivo* (27).

We directly uncovered IsrR mRNA targets by using MS2-affinity purification coupled with RNA sequencing (MAPS) (43). MAPS enriched for mRNA targets coding for proteins involved in cellular respiration, heme biosynthesis, and the oxidative stress response, all of which require Fe or generate an Fe-containing product. Of the nine previously validated IsrR targets, only one (*acnA*) was enriched in our MAPS analysis. It is unclear why we did not enrich for these targets, but it could be that IsrR has a lower affinity for these targets, or they were not highly expressed at the time point that we used for RNA purification. The finding that all enriched targets except four (*acnA, hemA*, *sodM,* and SAUSA300_0872) were absent from reported *in silico* analysis emphasizes the potential limitations of *in silico* tools. It also highlights the power of taking a direct approach, like MAPS, to explore the targetome of sRNAs. Of the 32 enriched mRNA discovered using MAPS, we successfully validated six targets *in vitro*. Genomic analysis identified three additional targets that we validated *in vitro*. Importantly, we demonstrated the physiological impact of IsrR regulation on dioxygen metabolism and heme synthesis. Future work is necessary to validate interactions for the remaining 26 transcripts identified by MAPS to determine if they are true IsrR targets.

**Fig 5:**
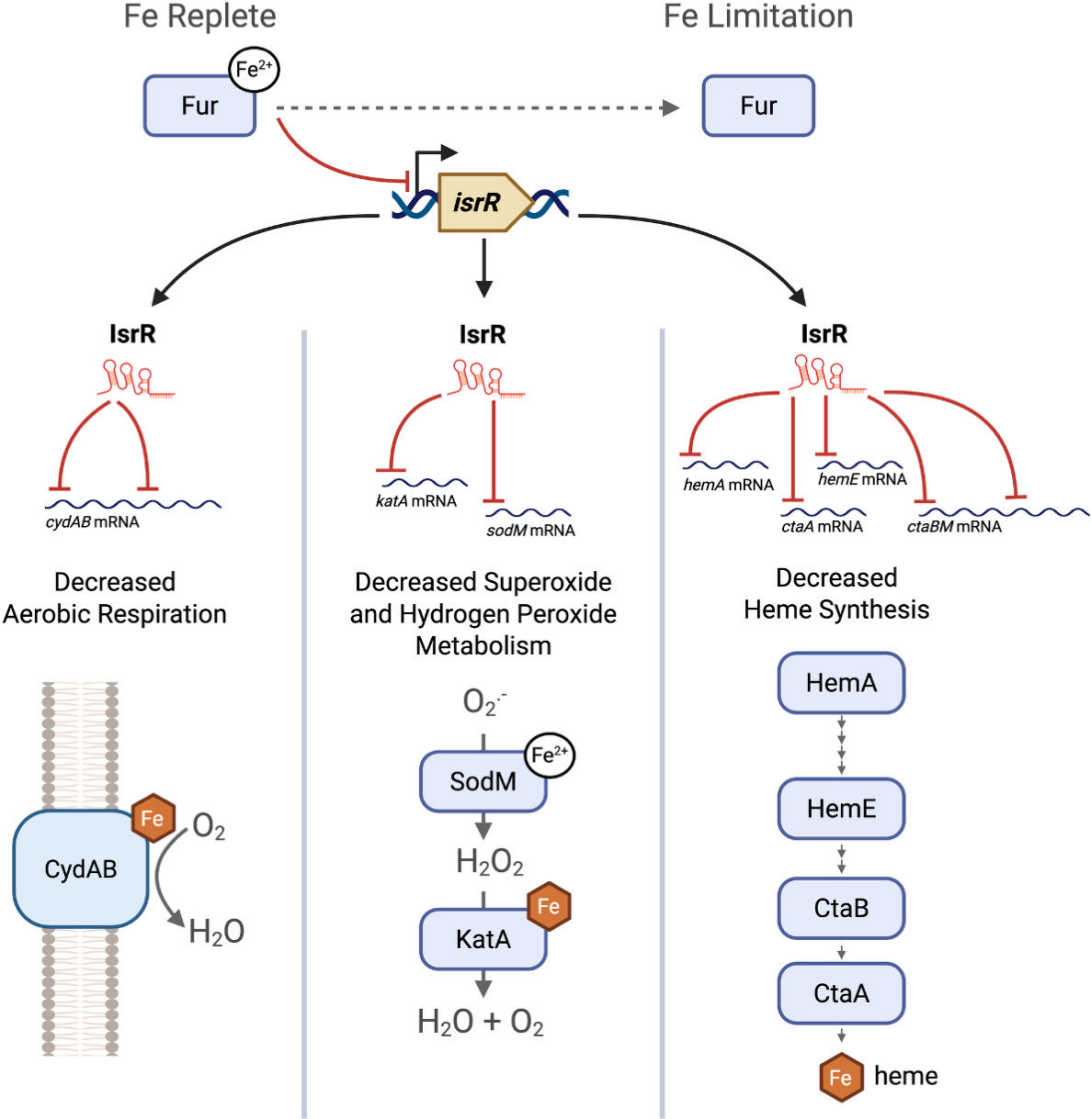
Working model for IsrR function. Under iron-replete conditions, Fur-Fe(II) represses *isrR* expression. Under iron-limiting growth conditions, Fur repression is alleviated, and *isrR* is expressed. IsrR binds to and represses the expression of mRNAs that code for proteins involved in aerobic respiration (CydAB), hydrogen peroxide, and superoxide detoxification (SodM, KatA), and heme synthesis (HemA, HemE, CtaA, CtaB, and CtaM).

Contrary to coagulase-negative staphylococci, *S. aureus* contains two superoxide dismutases (SodA and SodM). The presence of the two SODs allows for increased flexibility in mitigating oxidative stress while navigating metal ion limitation. Under Mn limiting conditions, the sRNA RsaC is processed from the 3’ UTR of the *mntABC* transcript. RsaC represses the translation of *sodA* and indirectly promotes SodM synthesis, mediating the switch from the Mn-dependent SodA to the cambialistic SodM during Mn scarcity (44). We demonstrated that IsrR bound to the *sodM* transcript *in vitro,* resulting in *sodM* translational repression and decreased SodM activity. The finding that under Fe-limiting conditions, IsrR is expressed and represses the expression of SodM (Fig. 5) reveals a second mechanism of *S. aureus* SOD regulation by metal-responsive sRNAs.

MAPS analysis enriched various mRNAs coding for heme synthesis and maturation proteins (GtrR/HemA, CtaM, and CyoE/CtaB). IsrR directly bound the *ctaA* and *hemE* transcripts. Supporting a role for IsrR in regulating heme synthesis, a *fur* mutant had an *isrR*-dependent decrease in heme synthesis. To our knowledge, regulation of heme biosynthesis in *S. aureus* has been limited to the first commitment step catalyzed by GtrR (HemA). The abundance of GtrR is negatively regulated by heme status and the membrane protein HemX (45). GtrR is also regulated by phosphorylation and dephosphorylation of the serine/threonine kinase and phosphatase pair Stk1 and Stp1, which regulate heme synthesis in response to growth status (46). In addition to heme concentration and growth status, heme synthesis is translationally regulated during iron limitation by IsrR (Fig. 5).

IsrR is a member of the family of metal-responsive sRNAs, which were initially revealed upon the discovery of RyhB in *Escherichia coli* (47). Like *isrR, ryhB* is expressed under low Fe conditions and decreases the expression of expendable Fe-containing proteins. RyhB also functions to re-establish Fe homeostasis by promoting the production of Fe-uptake siderophores and coordinating the biogenesis of iron-sulfur (Fe-S) cofactors. Chareyre and Mandin reviewed the RyhB targets, which comprised 56 confirmed or very likely mRNA targets coding for 143 proteins (48). Like IsrR, RyhB mediates the regulation of heme synthesis (*hemH, hemB*), catalase (*katG*), aerobic respiration (*cydAB*), and an Fe-requiring superoxide dismutase (*sodB*). Considering the plethora of RyhB mRNA targets and their similarities to IsrR, it is likely that IsrR is controlling additional mRNAs associated with Fe-using processes. RyhB homologs play a role in virulence by promoting the expression of virulence-associated traits such as acid stress resistance and biofilm formation. Remarkably, although RyhB-like sRNAs do not share sequence similarities, they all belong to the Fur regulon and regulate expression of a similar targetome. Future studies will focus on whether the virulence phenotypes of an *isrR* mutant result from an inability of *S. aureus* to mount an effective Fe-sparing response and/or if IsrR has a direct role in promoting the expression of virulence factors, as suggested by our MAPS experiments.

Many of the IsrR targets we examined are essential for full *S. aureus* pathogenesis. Immune cells target pathogens by generating toxic reactive oxygen species, which *S. aureus* detoxifies with enzymes like IsrR-targets KatA and SodM. Although a *katA* strain was not attenuated for virulence in a murine abscess infection model, it was required for nasal colonization (12, 49). Catalase expression is associated with virulent *S. aureus* isolates and increased mouse lethality, implying that KatA is a significant virulence factor (50, 51). SodM is critical for *S. aureus* infection during calprotectin-mediated Mn limitation, and a *sodM* mutant has reduced bacterial burden during systemic infection (14).

Another key aspect that contributes to the prevalence of *S. aureus* is its metabolic adaptability, showcased by a branched aerobic respiratory chain with two heme-dependent terminal oxidases in CydAB (*bd* type) and QoxABCD (*aa_3_* or *bo_3_* type). Inhibiting respiration by inactivating heme synthesis results in decreased host colonization (30). A lack of heme for terminal oxidases also leads to a severe growth defect, resulting in a small colony variant (SCV) phenotype. SCVs are clinically relevant because they can cause persistent infections and are more resistant to some antibiotics (52). We show IsrR directly interacts with the *hemA, ctaA,* and *ctaB* transcripts, and their individual inactivation is associated with virulence attenuation (30, 53, 54). IsrR also targeted both genes of the *cydAB* operon, which code for cytochrome *bd*. An *S. aureus cydB* mutant has decreased colonization in the heart under systemic infection (30). As the respiratory chain is likely a source of H_2_O_2_, decreasing electron flux through respiratory pathways may reduce ROS-related stress, benefiting iron-starved cells where the synthesis of SodM and KatA is reduced. Although individually important for full pathogenesis, IsrR targeting heme synthesis, oxidative stress, and aerobic respiration emphasizes the importance of managing Fe ion homeostasis during host colonization and pathogenesis.

This work has led to a working model for IsrR function (Fig. 5). Under Fe-limiting conditions, Fur repression of *isrR* is relieved. Expressed IsrR associates with and decreases the expression of the *cydA*, *cydB*, *sodM*, *katA*, *hemA*, *hemE*, *ctaA*, *ctaB,* and *ctaM* transcripts. This results in lower rates of dioxygen respiration, superoxide and hydrogen peroxide metabolism, and decreased heme synthesis. These findings demonstrate how *S. aureus* leverages critical processes such as metabolism and the oxidative stress response to adapt to iron scarcity. We describe how iron levels are a third stimulus, along with the NAD^+^/NADH ratio and electron flux through the respiratory chain, that regulates expression of the terminal oxidases in *S. aureus* (55, 56). We hypothesize that by decreasing non-essential Fe usage, *S. aureus* spares iron for processes essential for survival or pathogenesis. We previously demonstrated that Fe-S cluster synthesis is essential in *S. aureus*, but it is unclear which essential protein(s) demand this prosthetic group (57). Some proteins that may be prioritized upon Fe limitation include adenine DNA glycosylase MutY, ATP-dependent helicase/deoxyribonuclease subunit B AddB, and ribonucleotide reductase subunit NrdG, which bind Fe-S clusters and have roles in base excision repair, double-strand break repair, and deoxyribonucleotide biosynthesis, respectively (58–60).

## MATERIALS AND METHODS

### Chemicals, bacterial strains, and growth conditions

Unless specified, the *S. aureus* strains used in this study (Table 3) were isogenic and constructed in the community-associated *S. aureus* methicillin-resistant (MRSA) strain USA300_LAC that was cured of the native plasmid pUSA03 that confers erythromycin resistance (61). Unless otherwise stated, all *S. aureus* strains were grown at 37°C in tryptic soy broth (TSB) (MP Biomedicals). Solid tryptic soy agar (TSA) was generated by adding 1.5% (wt/vol) agar (VWR). Liquid cultures were shaken at 200 rpm. Unless stated otherwise, cells were cultured in 10 mL capacity culture tubes containing 2.0 mL of liquid medium. The optical densities of cultures were measured at 600 nm (OD_600_).

**TABLE 3:** *S. aureus* strains used in this study

Name	Genotype	Reference(s)
USA300_LAC		
JMB1100	USA300_LAC	(62)
JMB10842	Δ*fur::tetM*	(22)
JMB11292	Δ*isrR*	(22)
JMB11293	Δ*isrR* Δ*fur::tetM*	(22)
JMB4536	*hemB::Tn*	(55, 63)
JMB8563	Δ*acnA::tet*	(64)
JMB2078	*katA::Tn*	(65)
JMB14125	*sucA::Tn*	(63); this study
JMB1886	pLL39	(66)
JMB11448	*proC::Tn attB::*pLL39	(22)
JMB11449	*fur_E11stop_ proC::Tn attB::*pLL39	(22)
JMB11392	Δ*isrR fur_E11stop_ proC::Tn attB::*pLL39	(22)
JMB11393	Δ*isrR fur_E11stop_ proC::Tn attB::*pLL39_*isrR*	(22)
JMB11397	Δ*isrR attB::*pLL39	This study
JMB11398	Δ*isrR attB::*pLL39_*isrR*	This study
HG001		
DL056	HG001	(67)
DL566	Δ*isrR*	This study
DL526	*MS2-isrR*	This study

Antibiotics were added at the following final concentrations: 100 µg mL^−1^ ampicillin (Amp); 10 µg mL^−1^ chloramphenicol (Cm) to select for plasmids and 3.3 µg mL^−1^ Cm to maintain plasmids (TSB-Cm). Protein concentrations were determined using Bradford reagent (Bio-Rad Laboratories Inc., Hercules, CA). Unless stated otherwise, all chemicals were purchased from Sigma-Aldrich (St. Louis, MO). DNA primers were purchased from IDT and are listed in Table S1.

### Plasmid and strain construction

The restriction minus strain *S. aureus* RN4220 was used for transformations (68), and transductions were conducted using bacteriophage 80α (69). *Escherichia coli* 5-alpha (NEB) cultured in lysogenic broth was used for plasmid preparation. Plasmids used are listed in Table S2. Quick Ligase, restriction enzymes, and the HiFi DNA Assembly kit were purchased from New England Biolabs (NEB). All bacterial strains were PCR or sequence verified before use. Plasmid DNA and PCR products were sequenced by Azenta Life Sciences (South Plainfield, NJ) or Eurofins Genomics.

The pOS_P*_lgt_*_*sodM_gfp,* pOS_P*_lgt_*_*cydA_gfp,* pOS_P*_lgt_*_*hemA_gfp,* and pOS_P*_lgt_*_*ctaB_gfp* translational reporters were generated using the pOS_P_*lgt*_ digested with NdeI. The *sodM* insert of the pOS_P*_lgt_*_*sodM_gfp* was created using the plgt_SODM FWD and the SODM_gfp REV primer pair with JMB1100 as template DNA. The *gfp* insert of the pOS_P*_lgt_*_*sodM_gfp* was created using the sodM_GFP FWD and gfp_plgt rev primer pair with the pOS_*saeP1_gfp* plasmid (70) as template DNA. The *cydA* insert of the pOS_P*_lgt_*_*cydA_gfp* was created using the plgt_CYDA FWD and CYDA_gfp REV primer pair and JMB1100 as template DNA. The *gfp* insert of the pOS_P*_lgt_*_*cydA_gfp* was created using the cyda_GFP FWD and gfp_plgt rev primer pair with the pOS_*saeP1_gfp* plasmid as template DNA. The *hemA* insert of the pOS_P*_lgt_*_*hemA_gfp* was created using the plgt_HEMA and HEMA_gfp REV primer pair and JMB1100 as template DNA. The *gfp* insert of the pOS_P*_lgt_*_*hemA_gfp* was created using the hema_GFP FWD and gfp_plgt rev primer pair with the pOS_*saeP1_gfp* plasmid as template DNA. The *ctaB* insert of the pOS_P*_lgt_*_*ctaB_gfp* was created using the plgt_CTAB FWD and CTAB_gfp REV primer pair and JMB1100 as template DNA. The *gfp* insert of the pOS_P*_lgt_*_*ctaB_gfp* was created using the ctab_GFP FWD and gfp_plgt rev primer pair with the pOS_*saeP1_gfp* plasmid as template DNA.

### MS2-affinity purification coupled with RNA sequencing (MAPS)

MAPS experiments were performed in HG001 laboratory strain. The pMAD vector was used to both delete the *isrR* gene and chromosomally fuse the MS2 aptamer to the 5’ end of the *isrR* gene (Table S2). Briefly, growth at restrictive temperature (44°C) was followed by subcultures at 28°C in order to promote double crossover (71). The expression and stability of MS2-*isrR* were checked by Northern blot (see below; Fig. S1).

Overnight cultures in BHI were diluted 50-fold in 50 mL fresh medium (250 mL flasks) and grown with shaking at 37°C (180 rpm). Crude extracts from MS2-*isrR* and Δ*isrR* (control) cells were harvested (A) after induction by 250 µM 2,2′-dipyridyl (DIP) for 15 min when cells reached an OD_600_ = 1 or (B) after 6 h of growth in Chelex-100 (Sigma-Aldrich) treated RPMI (NRPMI) medium supplemented with 1 mM MgCl_2_ and 100 µM CaCl_2_.

MS2-affinity purifications were performed as described in Mercier et al. (29) in duplicates. The assessments of RNA quantity and quality were performed using Qubit fluorometric quantification and the 2100 Bioanalyzer instrument (Agilent). RNA samples were ribo-depleted using QIAseq FastSelect rRNA removal kit (QIAGEN), and cDNA libraries were prepared using NEBNext Ultra II directional RNA library prep kit for Illumina (NEB). cDNA libraries were sequenced using NextSeq 2000 system (Illumina). RNA-seq analysis was performed according to Lalaouna et al. (43) using DESeq2. Putative targets presented in Tables 1 and 2 were selected according to the following parameters: fold change (FC) >2 and *P*-value < 0.05.

### Northern blot analysis

Samples were harvested before and after MS2-affinity purification. Bacterial pellets were resuspended in RNA Pro Solution (FastRNA Pro Blue kit) and lysed using the FastPrep homogenizer (MP Biomedicals). Total RNA extraction was then performed following the manufacturer’s recommendations. Samples were loaded onto a 1% agarose gel containing 25 mM guanidium thiocyanate (Sigma-Aldrich). RNA was then transferred to a Hybond *N*+ nitrocellulose membrane (GE Healthcare Life Sciences), subsequently hybridized with IsrR- and 5S rRNA-specific digoxygenin (DIG)-labeled probes (Table S1), obtained using the DIG RNA Labeling Kit (Roche). Anti-digoxigenin-AP, Fab fragments, and CDP-Star (Roche) were used for luminescence detection. The results represent two independent experiments.

### Translational reporter assays

Overnight cultures of *S. aureus* strains containing a plasmid-based translational reporter were cultured overnight in 5 mL TSB supplemented with 3.3 µg mL^−1^ chloramphenicol (TSB-Cm) in 25 mL culture tubes at 37°C with shaking. Cultures were then diluted to an OD_600_ of 0.05 in triplicate into 5 mL TSB-Cm in 25 mL culture tubes and incubated at 37°C with shaking for 16 h. Optical density (OD_600_) and GFP fluorescence (excitation 485 nm and emission 520 nm) were measured in microtiter plates using a Varioskan Lux plate reader (Thermo Scientific).

### Electrophoretic mobility shift assays

Transcription start sites were determined according to Koch et al. (72). PCR fragments containing T7-*ctaA* (from −124 to +513), T7-*ctaB* (from −27 to +751), T7-*hemA* (from −28 to +691), T7-*hemE* (from −36 to +797), T7-*cydA* (from −42 to +781), T7-*cydB* (from −172 to +725), T7-*ctaM* (full length, from −207 to +462), T7-*katA* (from −42 to +392), and T7-*sodM* (full-length, from −28 to +651) were used as DNA template for *in vitro* transcription with T7 RNA polymerase. T7-*sodA* (full-length, from −28 to +600) and T7-*isrR* (full-length) were cloned into *pJET1.2/blunt* plasmid (ThermoFisher). T7 transcription was performed using XbaI and/or XhoI-digested plasmids as a template. RNAs were finally purified and radiolabeled when required (44).

5’-radiolabeled IsrR (20,000 cpm/sample, concentration <1 pM) and above-mentioned cold RNAs were separately denatured at 90°C in the buffer GR- (20 mM Tris-HCl pH 7.5, 60 mM KCl, 40 mM NH_4_Cl, 3 mM DTT), cooled 1 min on ice, and incubated at room temperature for 15 min in the presence of 10 mM MgCl_2_. Renatured RNAs were then mixed and incubated at 37°C for 15 min. Finally, the samples were loaded on a 6% polyacrylamide gel under non-denaturing conditions (300 V, 4°C). Gels were exposed to Fuji X-ray films and developed in an Optimax X-ray film processor. The results are representative of two independent experiments.

### Catalase assay

Catalase activity was monitored spectrophotometrically as reported elsewhere (65, 73). Bacterial strains were inoculated from a plate into 10 mL glass culture tubes containing 2 mL of TSB and cultured overnight at 37°C with shaking. Overnight cultures were diluted to an OD_600_ of 0.05 in triplicate in 2 mL of TSB, and the cells were cultured at 37°C, shaking for 16 h. One mL of cells was removed, pelleted by centrifugation, and washed twice with PBS. Cells were resuspended in 100 µL of lysis buffer (50 mM Tris, 150 mM NaCl, pH 7.4) containing 20 µg each of DNase I (Invitrogen) and lysostaphin (AMBI). After 1 h of incubation at 37°C, cells and cell debris were pelleted by centrifugation, and cell-free lysates were removed. The 1 mL assay reaction mixture contained 50 µM potassium phosphate buffer, pH 7.4, and 5 µL of cell-free lysate. The assay was initiated by the addition of 1.67 mM hydrogen peroxide. The decomposition of hydrogen peroxide was followed at 240 nM (ε = 43.6 M^−1^ cm^−1^) for 2 min (74). The protein concentration of the cell-free lysate was determined by Bradford assay (Bio-Rad).

### Hydrogen peroxide sensitivity assay

Sensitivity to Hydrogen Peroxide was determined as described previously (66). Bacterial strains were inoculated in triplicate into 10 mL glass culture tubes containing 2 mL of TSB and cultured overnight at 37°C with shaking. Overnight cultures were diluted to an OD_600_ of 0.05 in triplicate in 2 mL of TSB, and the cells were cultured at 37°C, shaking for 16 h. One milliliter of cells was pelleted by centrifugation and resuspended in PBS. The one mL reaction was carried out in 10 mL glass tubes, where cells were diluted to an OD_600_ of 0.7, and H_2_O_2_ (200 µM or 500 µM) was added. Samples were incubated at room temperature for 1 h. Subsequently, 50 µL of the reaction was quenched in 1 mL of PBS containing 1,300 units of bovine catalase. Cells were subsequently serially diluted and spot-plated on TSA.

### Heme quantification and sample preparation

The extraction and quantification of heme were performed as reported previously with some minor adjustments (46). Overnight cultures of *S. aureus* strains were grown in 50 mL TSB in 250 mL flasks at 37°C with shaking. After 16 h of growth, the OD_600_ of the cultures was recorded, and the culture was pelleted by centrifugation. Cell pellets were resuspended in 1 mL digestion buffer (PBS + 10 mM MgCl_2_ +40 µg/mL lysostaphin), transferred to 15 mL conical tubes, and incubated for 1 h. Prior to incubation, a 50 µL aliquot was transferred to a 2 mL screw cap tube containing 50 µL lysing matrix B for protein quantification. After digestion, aliquots were diluted in 200 µL PBS + 2% IGEPAL and disrupted by bead beating for 45 s at 4.5 m/s in an MP FastPrep bead beater. Tubes were centrifuged at 13,000 × 10 min, and the protein content of the supernatant was measured by Bradford assay (Bio-Rad). Digested samples for heme extraction were sonicated for 10 s at 100% using a Vibra-Cell sonicator (SONICS).

Heme was extracted from samples with 5 mL ethyl acetate +0.1% trichloroacetic acid (TCA). Samples were turned end over end at RT for 30 min, followed by centrifugation at 12,000 × *g* for 15 min. Four milliliters of the organic (top) layer were transferred to a glass tube and dried using a Centrivap Concentrator (LABCONCO). Samples were dissolved in 200 µL methanol and analyzed on an Agilent 1100 Series HPLC.

For quantification, 50 µL of sample or hemin standard (0, 1, 2.5, 5, 7.5, 10, 15, and 25 µM in MeOH) was separated using a Supelco Ascentis (C18 25 cm × 4.6 mm, 5 µm) column with a Phenomenex SecurityGuard (C18 cartridge 3.2 × 8 mm) guard column in place. Solvent A was 0.1% TCA in water, whereas solvent B was 0.1% in acetonitrile. The flow rate was set at 1.0 mL min^−1^ at room temperature for a run time of 27 min using the following successive linear gradient settings for run time in minutes vs B: 0.0, 25%; 1.0, 25%; 20.0, 100%; 23.0, 100%; 23.01, 25%; and 27.0, 25%. Absorbance was detected at 380, 398, and 411 nm. Under these conditions, heme elutes around 9 min, and we used 398 nm absorbance for quantification.

### Growth assays

Bacteria were cultured overnight in 5 mL RPMI with 1% casamino acids supplemented with 1 mM MgCl_2_, 100 µM CaCl_2_, 1 µM ZnSO_4_, and 1 µM FeSO_4_ (supplemented NRPMI) in 15 mL conical tubes on a roller drum at 37°C. These cultures were diluted 1:100 into 100 µL of culture medium in a 96-well round-bottom plate and incubated at 37°C with shaking at 180 rpm. The culture medium was the same as the overnight medium, with or without 0.1 µM paraquat (PQ). For growth on TSB medium, overnight cultures were diluted to an OD_600_ of 0.05, and 200 µL of culture was added to a 96-well microtiter plate. Culture optical density (OD_600_) was read using a BioTek 808E visible absorption spectrophotometer with continuous shaking and incubated at 37°C.

### SOD activity assay

Individual superoxide dismutase activity was assayed using a gel-based nitro blue tetrazolium assay conducted as previously described (14). Bacteria were grown in supplemented NRPMI as described for the growth assays with or without 1 µM FeSO_4_ and 100 µM 2,2-dipyridyl and were harvested at an OD_600_ of approximately 0.25. Collected cells were resuspended in 0.5 mM KPO_4_ buffer, pH 7.8, with 0.1 mM EDTA. The bacteria were lysed using a bead beater (MP Biomedical) and centrifuged to remove insoluble material and whole cells. The protein concentrations in the cell lysates were determined via BCA assay (Pierce). Eight micrograms of lysate was loaded in each well of a 10% native polyacrylamide gel. The gels were incubated in 50 µM KPO_4_ buffer, pH 7.8, 1 mM EDTA, 0.25 mM nitro blue tetrazolium chloride, and 50 µM riboflavin, and subsequently exposed to light.

### Qualitative measurement of membrane potential

Overnight cultures were grown in 2 mL TSB in 10 mL culture tubes at 37°C with shaking. Strains were diluted 1:100 into 2.5 mL TSB and incubated at 37°C with shaking for 6 h. After incubation, 2 mL of cells were pelleted, washed with 0.5 mL PBS, pH 7.4, and adjusted to an OD_600_ of 0.085 in PBS. To a black 96-well plate, 200 µL of cell suspension were added with 30 µM 3,3′-diethyloxacarbocyanine iodide (DiOC_2_(3)) +/− 5 µM carbonyl cyanide m-chlorophenylhydrazone (CCCP), followed by incubation at room temperature for 30 min. Red fluorescence (excitation of 450 nm, emission of 670 nm) and green fluorescence (excitation of 485 nm, emission of 520 nm) were measured using a Varioskan Lux plate reader (Thermo Scientific).

### Dioxygen consumption and media acidification assays

*S. aureus* strains were grown overnight in 5 mL TSB in 30 mL culture tubes at 37°C in a shaker (200 RPM). Strains were diluted 1:100 into 10 mL TSB in 125 mL flasks and incubated at 37°C with agitation for 4 h. The cultures were diluted to an OD_600_ of 0.025 in TSB prior to transfer of 200 µL to the wells of a Seahorse XF96 V3 PS cell culture microplate (Agilent). The Seahorse XF sensor cartridge (Agilent) was hydrated in a non-CO_2_ incubator with sterile water overnight and equilibrated in XF calibrant (Agilent) for 2 h prior to measurement. Measurements were taken for 15 cycles with a 3 min mix and 3 min measure cycle.

## Data Availability

Raw and processed data are available in the GEO database under accession number GSE296222.

## SUPPLEMENTAL MATERIAL

Supplemental material mbio.01415-25-s0001.pdf Supplemental figures and tables.

ASM does not own the copyrights to Supplemental Material that may be linked to, or accessed through, an article. The authors have granted ASM a non-exclusive, world-wide license to publish the Supplemental Material files. Please contact the corresponding author directly for reuse.
