# Supplementary material for: The iron-regulated small regulatory RNA IsrR modulates expression of genes utilized for dioxygen metabolism and heme synthesis in Staphylococcus aureus
Source: mBio. 2025 Oct 3;16(11):e01415-25. doi: 10.1128/mbio.01415-25 (PMC12607789; doi:10.1128/mbio.01415-25)
Supplement: Supplemental material — Supplemental figures and tables. [file mbio.01415-25-s0001.pdf]

Supplemental material for:

The iron-regulated small regulatory RNA IsrR modulates expression of genes utilized for dioxygen metabolism and heme synthesis in *Staphylococcus aureus*.

Gustavo Rios-Delgado[1], Riley McFarlane[3], Vincent Zheng[1], Jisun Kim[4], Dane Parker[4], Thomas Kehl-Fie[3], David Lalaouna[2,*], Jeffrey M. Boyd[1,*]

[1] Department of Biochemistry and Microbiology, Rutgers, the State University of New Jersey, New Brunswick, NJ, 08901, USA.

[2] Université de Strasbourg, CNRS, Architecture et Réactivité de l'ARN, UPR9002, Strasbourg, 67000, France

[3] Department of Microbiology and Immunology, Carver College of Medicine, University of Iowa, Iowa City, Iowa, 52242, USA.

[4] Department of Pathology, Immunology and Laboratory Medicine, Center for Immunity and Inflammation, Rutgers New Jersey Medical School, Newark, New Jersey, 07103, USA.

*To whom correspondence may be addressed:

David Lalaouna, RNA architecture and reactivity (UPR9002), Institut de Biologie Moléculaire et Cellulaire, 2 Allée Konrad Roentgen, Strasbourg, France, Telephone: +33 3 88 41 70 51, E-mail: d.lalaouna@ibmc-cnrs.unistra.fr

Jeffrey M. Boyd, Department of Biochemistry and Microbiology, Rutgers University, 76 Lipman Drive, New Brunswick, New Jersey, 08901, Telephone: (848) 932-5604, E-mail: jeffboyd@SEBS.rutgers.edu.

Supplemental Tables

Table S1: Oligonucleotides used in this study.

Primer Name	Sequence
plgt_SODM FWD	AATTGAGGTGAACATAATTTTATATAATTGGATATAACAAATAAA TAATAATTATTGCAA
SODM_gfp REV	AGTTCTTCTCCTTTGCTCATAAGTATATTCCTCCTTTTATGAAT ATACT
sodm_GFP FWD	ATAAAAGGAGGAATATACTTATGAGCAAAGGAGAAGAACT
plgt_CTAB FWD	AATACAATTGAGGTGAACATACATTATAGCAATAAATTAAATGT TATTTGTG
CTAB_gfp REV	AGTTCTTCTCCTTTGCTCATGCTCATAATCCCCCTCCTTA
ctab_GFP FWD	TAAGGAGGGGGATTATGAGCATGAGCAAAGGAGAAGAACT
plgt_CYDA FWD	ATACAATTGAGGTGAACATACACAATTTCATAGCGCATAATG
CYDA_gfp REV	AGTTCTTCTCCTTTGCTCATATCCATTTAAAAACATCACCTTTT T
cyda_GFP FWD	GGTGATGTTTTTAAATGGATATGAGCAAAGGAGAAGAACT
plgt_HEMA	ATACAATTGAGGTGAACATACACAATTGATAACATTTTTCAACG TTC
HEMA_gfp REV	AGTTCTTCTCCTTTGCTCATATGCATTTGAAACGCCCCCA
hema_GFP FWD	TGGGGGCGTTTCAAATGCATATGAGCAAAGGAGAAGAACT
Gfp_plgt rev	AAAGGGGGAAACACTACCCCCTTGTTTGGATCTTAGTGGTGG TGGTGGTGGTGGGAT
T7-*isrR*-For	TAATACGACTCACTATAGGGAAAATGATTATCAATACCAC
T7-*isrR*-Rev	AAACAAAAGCAGTAAACCTAAAGTGTCG
T7-*sodM*-For	TAATACGACTCACTATAGGGGTATATTCATAAAAGGAGGAAT
T7-*sodM*-Rev	AAAAAAGTGGAACACCTTGTAGATGC
T7-*katA*-For	TAATACGACTCACTATAGGGATGTATTAAATTATAATTATTATAAA TTG
T7-*katA*-Rev	GTGTTATTCCCTACTAAATCCCAG
T7-*ctaA*-For	TAATACGACTCACTATAGGGTTAGAAAATTTTAATAAGTAGAAT C
T7-*ctaA*-Rev	GTTTTGTTGCCAAATAACAGCAGC
T7-*ctaB*-For	TAATACGACTCACTATAGGGATATGAACAAATTTAAGGAGGG
T7-*ctaB*-Rev	CTAACACTACGAATACGACACC
T7-*hemA*-For	TAATACGACTCACTATAGGGATTGTTAGTATATATGGGGGC
T7-*hemA*-Rev	CATATTTCACTTGATGCTTTGCTGC
T7-*hemE*-For	TAATACGACTCACTATAGGGATATAGATAAGAATGATTTTAATTT AG
T7-*hemE*-Rev	CAATCTAGGCCTAATACATCAATCG
T7-cydA-For	TAATACGACTCACTATAGGGCATTGTGTTGTCATCAAAAAAAG
T7-cydA-Rev	CGAAATGCCATTCATAAGCTGC
T7-cydB-For	TAATACGACTCACTATAGGGCAGAAGCAGCAACACAAG
T7-cydB-Rev	CTTATACCAAACATCCACCAATAGTC
T7-ctaM-For	TAATACGACTCACTATAGGGTGTCGTATTCGTAGTG

T7-ctaM-Rev	TTAATGACCAAATGTTGCTTTAATCAGTG
IsrR-DIG-For<br>IsrR-DIG-Rev	TAATACGACTCACTATAGGGCAGTAAACCTAAAGTGTCG<br>GATTATCAATACCACATAGAACATCCC
5S-DIG-For	TAATACGACTCACTATAGGGGATTTGTCATTTGCCTGGC
5S-DIG-Rev	GTAAGTTATTTTGTCTGGTGGCTATAGC
MS2-isrR-UF-BamHI	CATAGGATCCGCGTCATTTGTACACCTCATATTACG
MS2-*isrR*-UR	CTGAAAAACGTACCCTGATGGTGTACGAACTACTATTATACATTAGTGAG
MS2-*isrR*-DF	GTACGTTTTTCAGACACCATCAGGGTCTGTTTGAAAATGATTATCAATACCACA
MS2-*isrR*-DR-NcoI	CATACCATGGCGAATCAAGACTTGGGAAATGGATTG
IsrR-For	CCATCATACTCTGTGTCCACATTG
IsrR-Rev	GTAATGCTTTATGAGTCAATGGAAACG
isrR-UF-XbaI	GCTCTAGAGCGTCATTTGTACACCTCATATTACG
isrR-UR	GGTTTACTGCGTAAATAAAACGCATGATTACG
isrR-DF	GTTTTATTTACGCAGTAAACCCTTACGAC
isrR-DR-XhoI	CGGCTCGAGCGAATCAAGACTTGGGAAATGGATTG

Table S2: Plasmids used in this study.

Name	Function	Reference
pOS-1-P_{lgt}	Genetic complementation	(1)
pOS-1-P_{lgt}_*cydA_gfp*	*cydA* translational reporter	This study
pOS-1-P_{lgt}_*sodM_gfp*	*sodM* translational reporter	This study
pOS-1-P_{lgt}_*hemA_gfp*	*hemA* translational reporter	This study
pOS-1-P_{lgt}_*ctaB_gfp*	*ctaB* translational reporter	This study
pJET-*sodA*	Generate *sodA* transcript	(2)
pJET-*isrR*	Generate *isrR* transcript	This study
pLL39	Genetic complementation	(3)
pLL39_*isrR*	*isrR* complementation	(4)
pMAD	Mutagenesis	(5)
pMAD-*MS2-isrR*	Insertion of MS2 sequence	This study
pMAD-Δ*isrR*	Deletion of *isrR* gene	This study

Supplemental Figures.

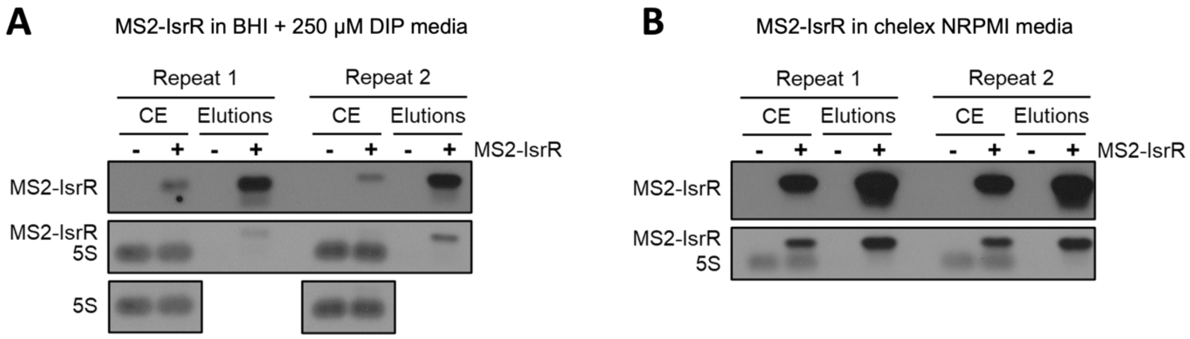

Supplemental Figure 1: MS2-IsrR construct validation. Northern blot analysis of MS2-IsrR RNA levels before (Crude extracts, CE) and after MS2-affinity purification (Elutions) using an IsrR-specific DIG probe. To induce iron starvation, MS2-*isrR* and Δ*isrR* cells were grown in (A) BHI medium + 250 µM DIP and (B) Chelex treated-RPMI medium supplemented with 1 mM $MgCl_2$ and 100 µM $CaCl_2$. Cells were harvested at OD_{600nm}≈1. 5S rRNA was used as loading control.

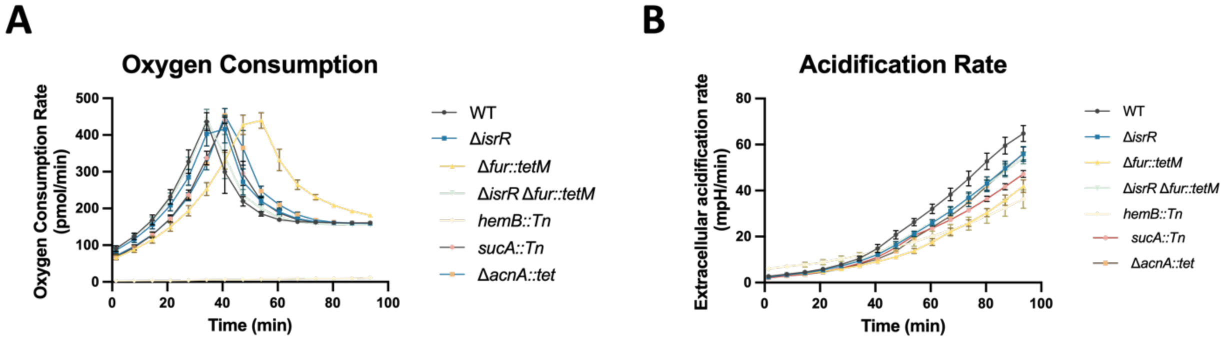

Supplemental Figure 2: IsrR impacts oxygen consumption and media acidification rate. **Panel A**. Time course of Oxygen Consumption Rate of wild type (JMB1100), Δ*fur::tet* (JMB10842), Δ*isrR* (JMB11292), Δ*isrR* Δ*fur::tet* (JMB11293), *sucA::Tn* (JMB14125), Δ*acnA::tet* (JMB8563), and *hemB::Tn* (JMB4536) strains cultured in Fe-replete TSB incubated at 37°C. **Panel B**. Time course of the Extracellular Acidification Rate of the strains in Panel A cultured in TSB and incubated at 37°C. Data points indicate experimental averages (n=8) and error bars indicate standard deviation.

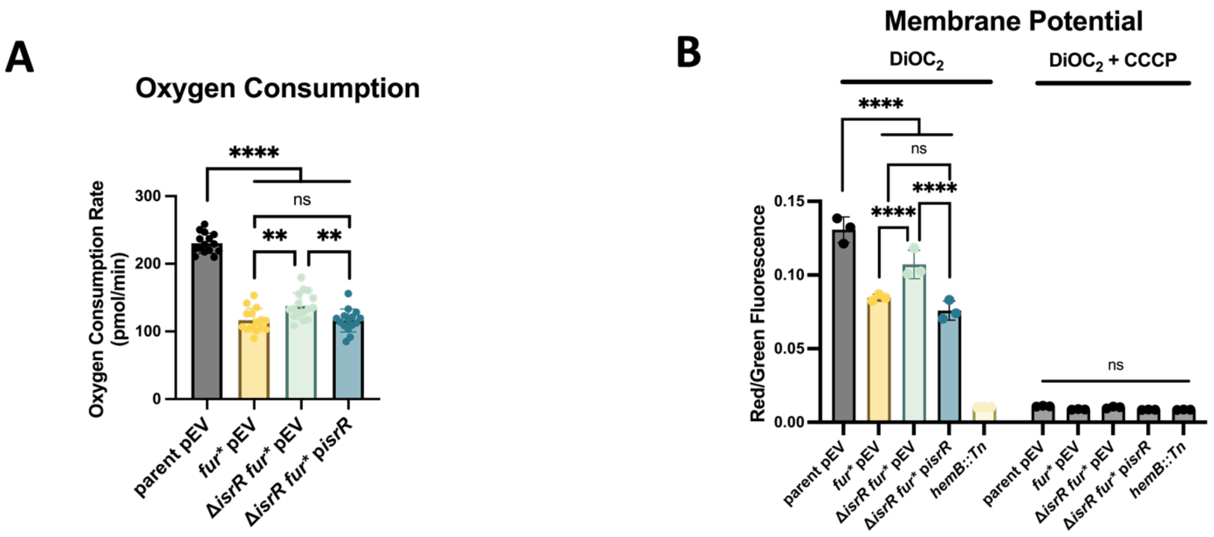

Supplemental Figure 3: IsrR impacts aerobic respiration, complementation of Δ*isrR* Δ*fur* phenotype. **Panel A**. Oxygen Consumption Rates of the *proC::Tn* pLL39 (parent pEV; JMB11448), *proC::Tn fur** pLL39 (*fur** pEV; JMB11449), *proC::Tn fur** Δ*isrR* pLL39 (Δ*isrR fur** pEV; JMB11392), and *proC::Tn fur** Δ*isrR* pLL39_*isrR* (Δ*isrR fur** p*isrR*; JMB11393) strains cultured in Fe-replete TSB incubated at 37°C cultured in TSB after 44 min incubation at 37°C. **Panel B**. Membrane potentials of the strains in Panel A and *hemB::Tn* (JMB4536) were measured using the fluorescent dye 3'3'-diethyloxacarbocyanine iodide ($DiOC_2$) with and without membrane decoupler carbonyl cyanide m-chlorophenylhydrazone (CCCP). Data points indicate experimental averages (panel A n=16 or panel B n=3) and error bars indicate standard deviation. An Ordinary one-way ANOVA (Panel A) or Two-way ANOVA (Panel B) followed by Tukey's multiple comparisons test was used to analyze the data, and ** denotes p-value<0.01, ****p-value<0.0001.

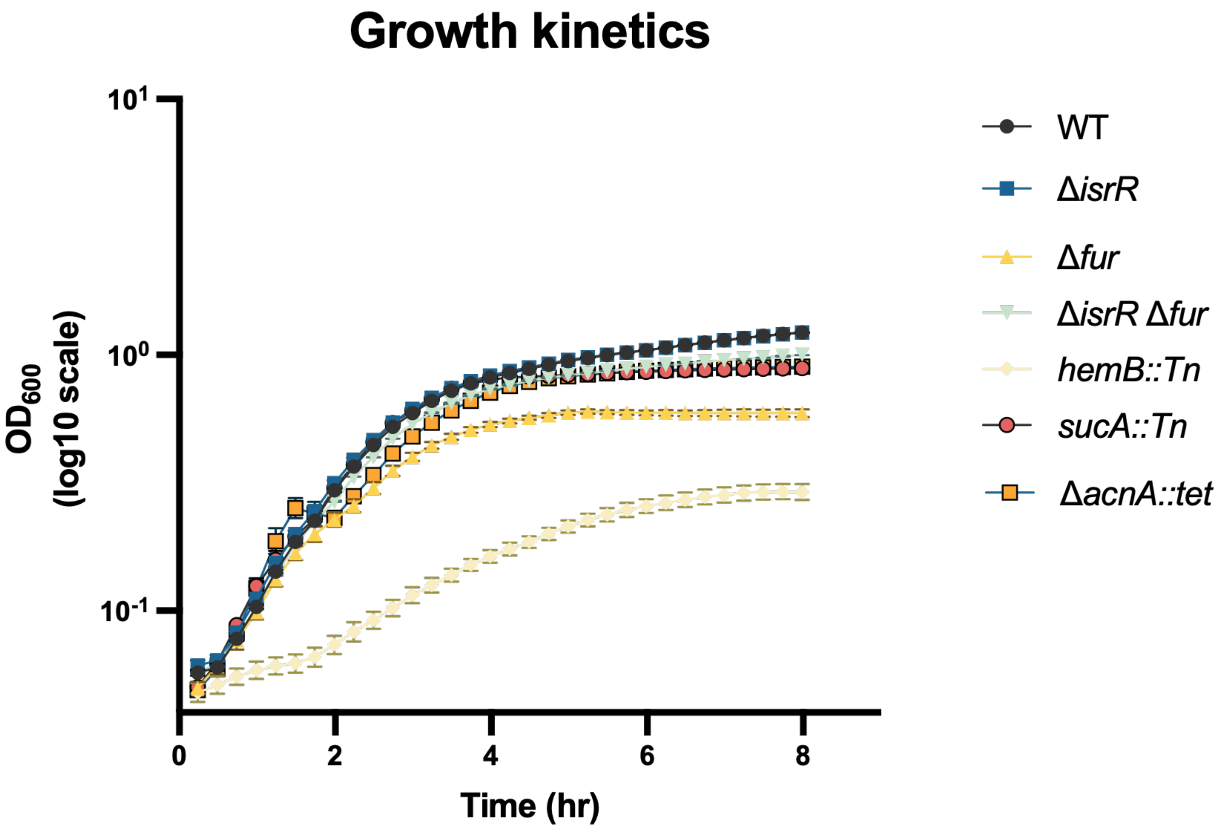

Supplemental Figure 4: Introduction of the Δ*isrR* mutation improves the growth of the Δ*fur* mutant. Culture absorbance (optical density at 600 nm (OD_{600})) was monitored for the wild type (WT; JMB1100), Δ*fur* (JMB10842), Δ*isrR* (JMB11292), Δ*isrR* Δ*fur* (JMB11293), *hemB::Tn* (JMB4536), *sucA::Tn* (JMB14125), and Δ*acnA::tet* (JMB8563) strains in TSB. Data points represent the averages of biological triplicates, and error bars indicate standard deviation, but in some cases are smaller than the symbols used.

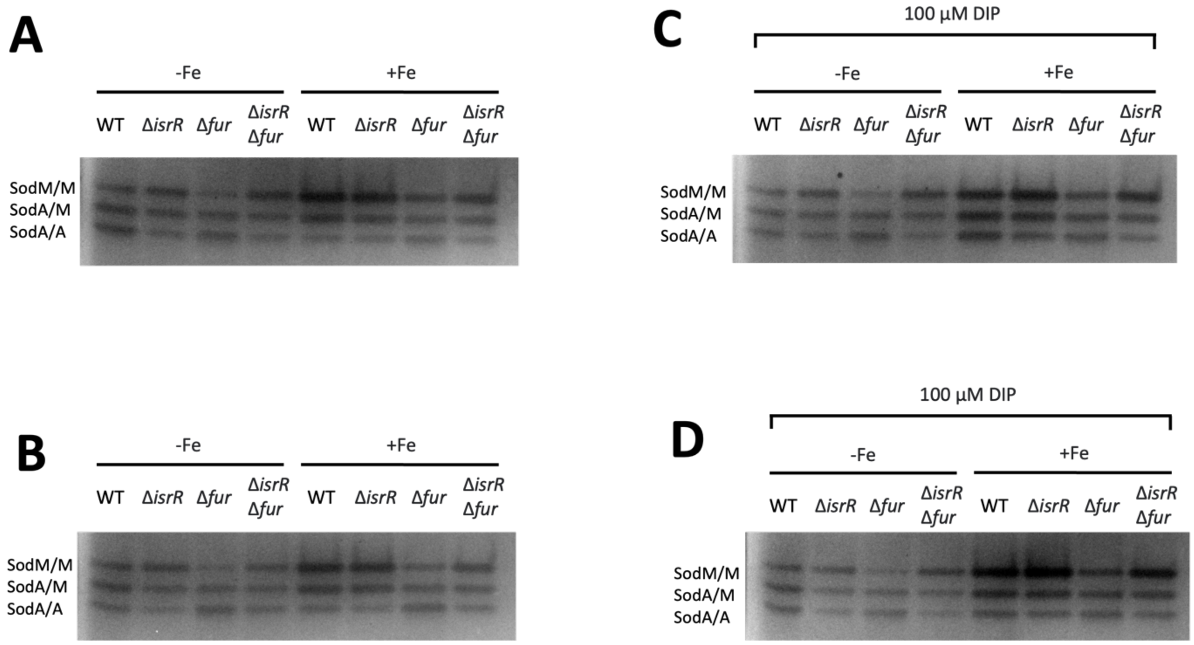

Supplemental Figure 5: Individual superoxide dismutase activity gels. **Panels A and B**. Superoxide dismutase activity gels of the wild type (JMB1100), Δ*fur::tet* (JMB10842), Δ*isrR* (JMB11292), Δ*isrR* Δ*fur::tet* (JMB11293) strains cultured in NRPMI with 1% Casamino acids, 1 mM $MgCl_2$, 100 μM $CaCl_2$, 1 μM $ZnSO_4$ media with and without 1 μM $FeSO_4$. **Panels C and D**. Superoxide dismutase activity gels of the strains in Panels A and B with the addition of 100 μM 2,2'-Dipyridyl (DIP). The top band corresponds to the SodM homodimer, the middle band to the SodA-SodM dimer, and the bottom band corresponds to the SodA homodimer.

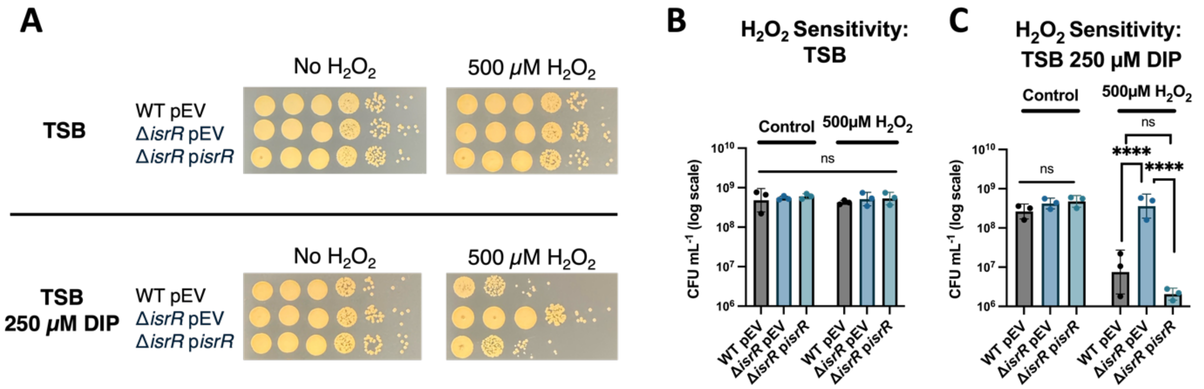

Supplemental Figure 6: IsrR impacts sensitivity to hydrogen peroxide. **Panel A**. Survival of the wild type with pLL39 (WT pEV; JMB1886), Δ*isrR* with pLL39 (Δ*isrR* pEV; JMB11397), and Δ*isrR* with pLL39_*isrR* (Δ*isrR* p*isrR*; JMB11398) after culture in TSB +/- 250 μM 2,2' dipyridyl (DIP) and treating with Hydrogen peroxide (H_2O_2). The reaction was quenched with catalase, bacteria were serially diluted, and 5 μL from each dilution was spot-plated on TSA plates to determine colony-forming units (CFU). The experiment was performed in triplicate, and an image of a representative experiment is displayed. **Panels B and C.** Quantification of CFU mL^{-1} of strains in Panel A. Data in panels B and C are reported as the average of the biological triplicates, and error bars indicate standard deviation. A two-way ANOVA followed by Tukey's multiple comparisons test was used to analyze the data in panels B and C, and **** denotes p-value<0.0001 while ns denotes not significant.

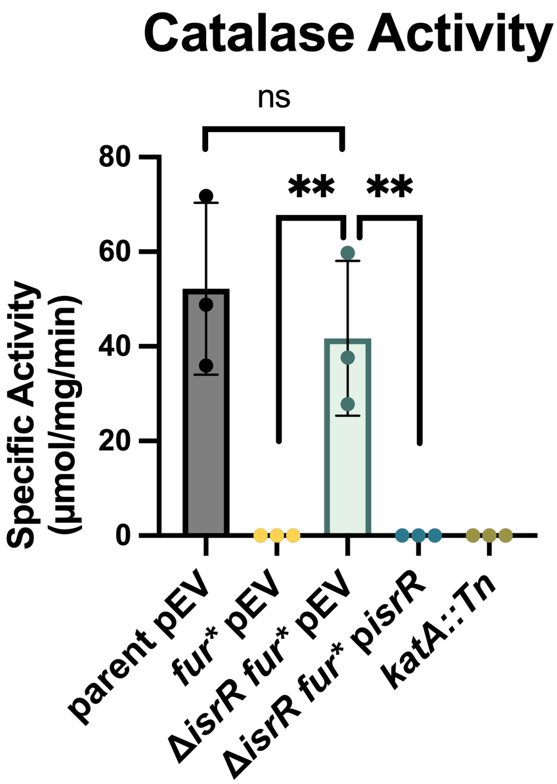

Supplemental Figure 7: IsrR represses catalase activity. The specific activity of catalase was quantified in cell-free lysates from the *proC::Tn* pLL39 (parent pEV; JMB11448), *proC::Tn fur** pLL39 (*fur** pEV; JMB11449), *proC::Tn fur** Δ*isrR* pLL39 (Δ*isrR fur** pEV; JMB11392), *proC::Tn fur** Δ*isrR* pLL39_*isrR* (Δ*isrR fur** p*isrR*; JMB11393), and *katA::Tn* (JMB2078) strains after culture in Fe-replete TSB incubated at 37°C. The data shown represent the average of biological triplicates with standard deviations. An Ordinary one-way ANOVA followed by Tukey's multiple comparisons test was used to analyze the data, and ** denotes p-value<0.01 and ns denotes non-significant.

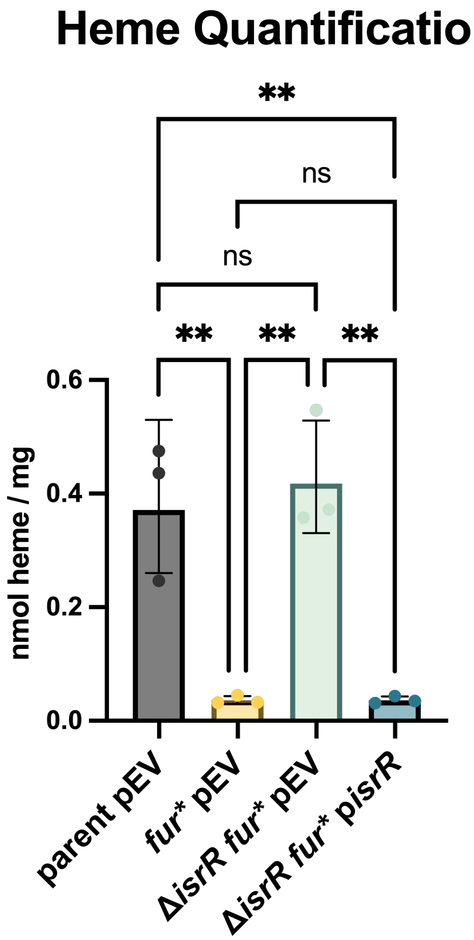

Supplemental Figure 8: IsrR represses heme biosynthesis. Heme was isolated and quantified from the *proC::Tn* pLL39 (parent pEV; JMB11448), *proC::Tn fur** pLL39 (*fur** pEV; JMB11449), *proC::Tn fur** Δ*isrR* pLL39 (Δ*isrR fur** pEV; JMB11392), and *proC::Tn fur** Δ*isrR* pLL39_*isrR* (Δ*isrR fur** p*isrR*; JMB11393) strains after culture in Fe-replete TSB. The data represent the average of biological triplicates with standard deviations displayed. An Ordinary one-way ANOVA followed by Tukey's multiple comparisons test was used to analyze the data, and ** denotes p-value<0.01 and ns denotes no significant differences.

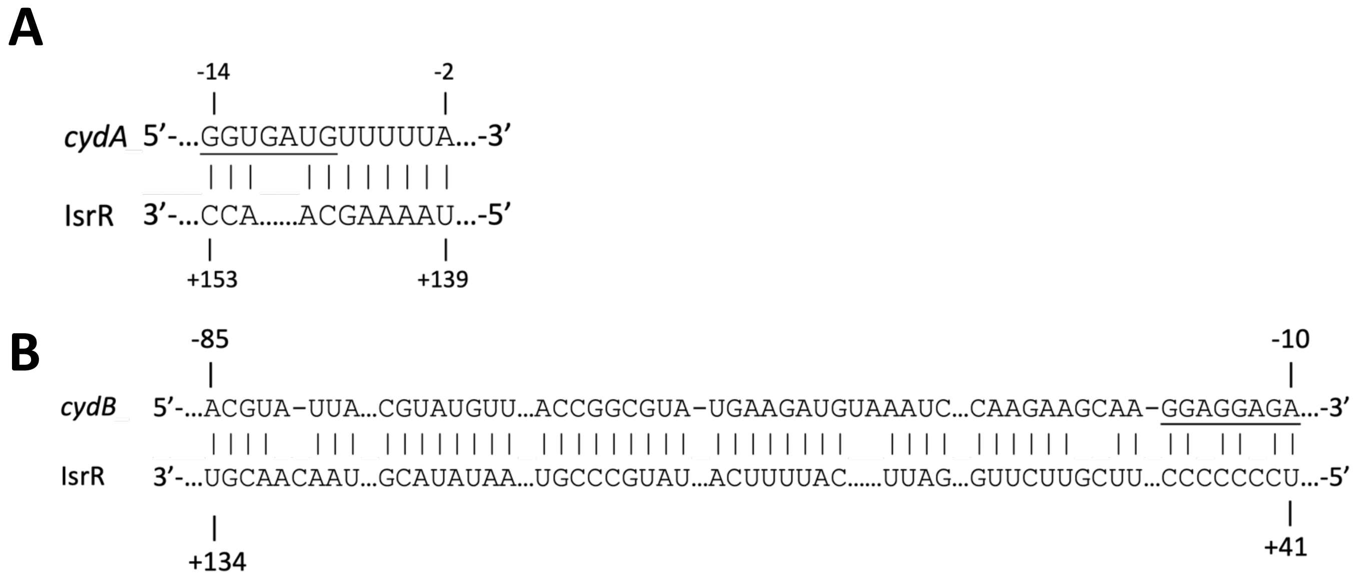

Supplemental Figure 9: Predicted interaction between IsrR and aerobic respiration mRNAs. **Panel A and B**. Predicted interaction between IsrR and *cydA* (Panel A, Energy= -2.4 kcal/mol) or *cydB* (Panel B, Energy= -3.12 kcal/mol) mRNAs by IntaRNA (6). The Shine-Dalgarno sequence is underlined.

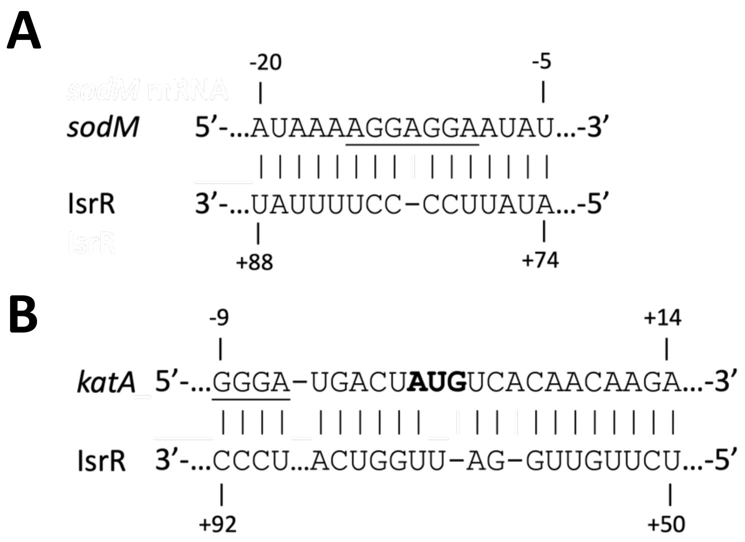

Supplemental Figure 10: Predicted interaction between IsrR and oxidative stress response mRNAs. **Panel A and B**. Predicted interaction between IsrR and *sodM* (Panel A, Energy= -8.24 kcal/mol) or *katA* (Panel B, Energy= -6.24 kcal/mol) mRNAs by IntaRNA (6). The start codon is bolded, and the Shine-Dalgarno sequence is underlined.

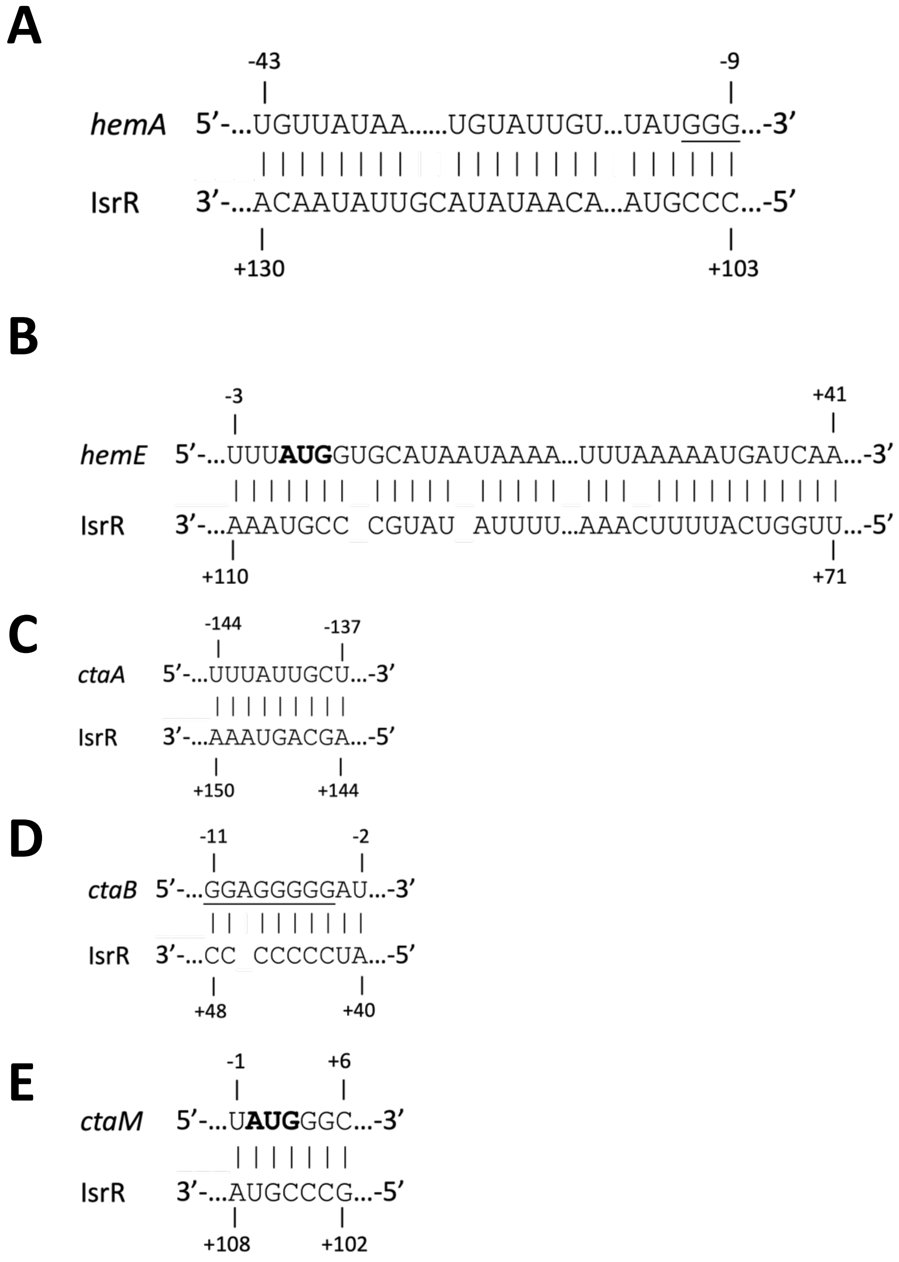

Supplemental Figure 11: Predicted interaction between IsrR and heme synthesis mRNAs. **Panels A-E**. Predicted interaction between IsrR and *hemA* (Panel A, Energy= -6.92), *hemE* (Panel B, Energy= -10.82 kcal/mol), *ctaA* (Panel C, Energy= -6.41 kcal/mol) *ctaB* (Panel D, Energy= -13.25 kcal/mol), *ctaM* (Panel E, Energy= -8.2 kcal/mol) mRNAs by IntaRNA (6). The start codon is bolded, and the Shine-Dalgarno sequence is underlined.

1. Bubeck Wardenburg J, Williams WA, Missiakas D. 2006. Host defenses against *Staphylococcus aureus* infection require recognition of bacterial lipoproteins. Proc Natl Acad Sci U S A 103:13831-6.
2. Lalaouna D, Baude J, Wu Z, Tomasini A, Chicher J, Marzi S, Vandenesch F, Romby P, Caldelari I, Moreau K. 2019. RsaC sRNA modulates the oxidative stress response of *Staphylococcus aureus* during manganese starvation. Nucleic Acids Res 47:9871-9887.
3. Luong TT, Lee CY. 2007. Improved single-copy integration vectors for *Staphylococcus aureus*. J Microbiol Methods 70:186-90.
4. Rios-Delgado G, McReynolds AKG, Pagella EA, Norambuena J, Briaud P, Zheng V, Munneke MJ, Kim J, Racine H, Carroll RK, Zelzion E, Skaar E, Bose JL, Parker D, Lalaouna D, Boyd JM. 2025. The *Staphylococcus aureus* non-coding RNA IsrR regulates TCA cycle activity and virulence. Nucleic Acids Res 53.
5. Arnaud M, Chastanet A, Debarbouille M. 2004. New vector for efficient allelic replacement in naturally nontransformable, low-GC-content, gram-positive bacteria. Appl Environ Microbiol 70:6887-91.
6. Mann M, Wright PR, Backofen R. 2017. IntaRNA 2.0: enhanced and customizable prediction of RNA-RNA interactions. Nucleic Acids Res 45:W435-W439.
